# Supplementary material for: Overexpression of the Phosphoserine Phosphatase-Encoding Gene (AtPSP1) Promotes Starch Accumulation in Lemna turionifera 5511 under Sulfur Deficiency
Source: Plants (Basel). 2023 Feb 23;12(5):1012. doi: 10.3390/plants12051012 (PMC10005638; doi:10.3390/plants12051012)
Supplement: Supplementary file 1 [file plants-12-01012-s001.zip › plants-2100139-supplementary.pdf]

Supplementary Materials

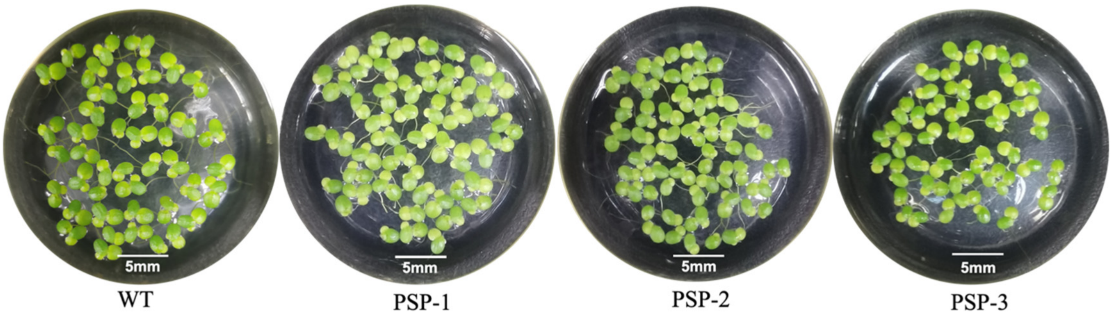

Figure S1. Phenotype of WT and three *AtPSP1* transgenic lines. The photoes were taken after WT and *AtPSP1* transgenic lines were cultivated after full nutrition condition (Datko) for 9 days.

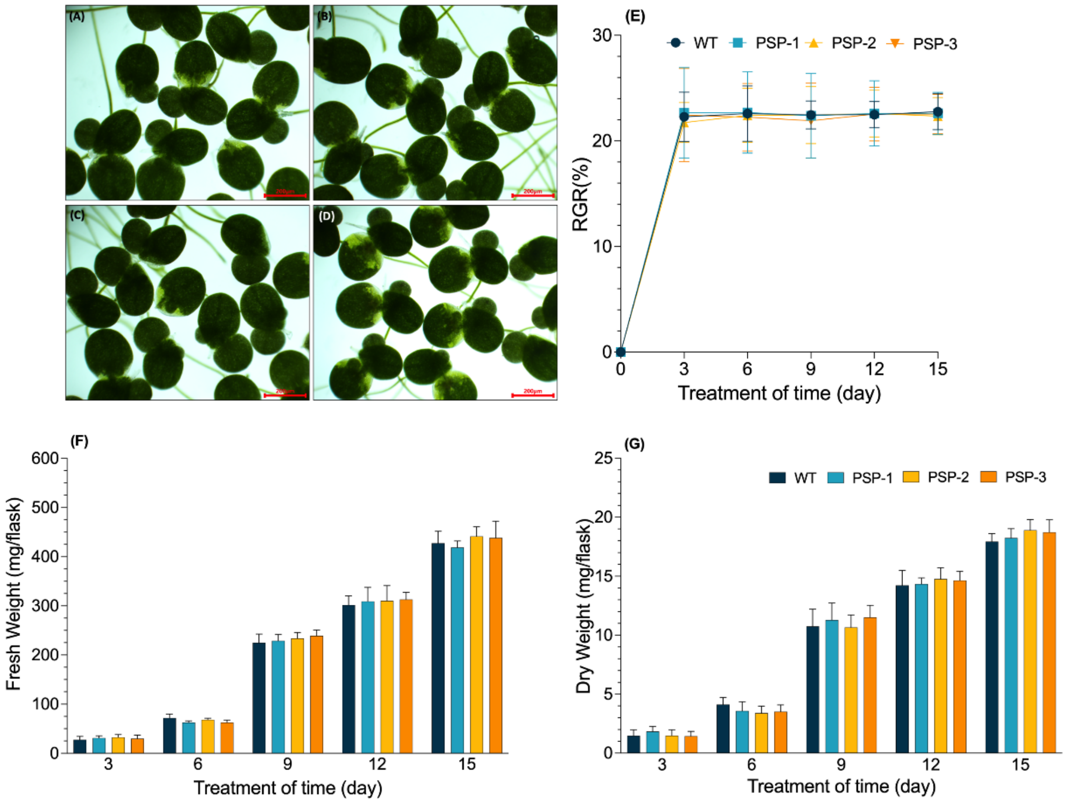

Figure S2. Analysis of phenotype, relative growth rate (RGR), fresh weight and dry weight in WT and three *AtPSP1* transgenic lines under Datko condition. **(A)** WT, **(B)** PSP-1, **(C)** PSP-2, **(D)** PSP-3 indicate the phenotype of WT and *AtPSP1* transgenic lines were cultivated under Datko condition for 9 days with dissecting microscope; **(E)** The RGR; **(F)** The fresh weight; **(G)** The dry weight. Each statistic is the mean ± standard error (n=9, from three independent experiments with three repeats of each).

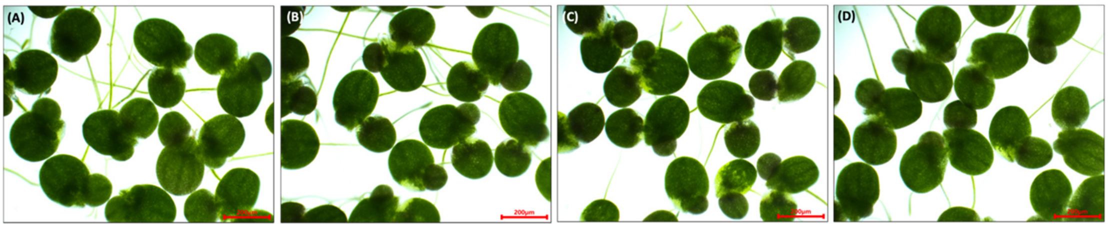

Figure S3. Phenotype of WT and three *AtPSP1* transgenic lines under sulfur-deficiency condition. **(A)** WT, **(B)** PSP-1, **(C)** PSP-2, **(D)** PSP-3 indicate the phenotype of WT and *AtPSP1* transgenic lines were cultivated under sulfur-deficiency condition for 9 days with dissecting microscope.

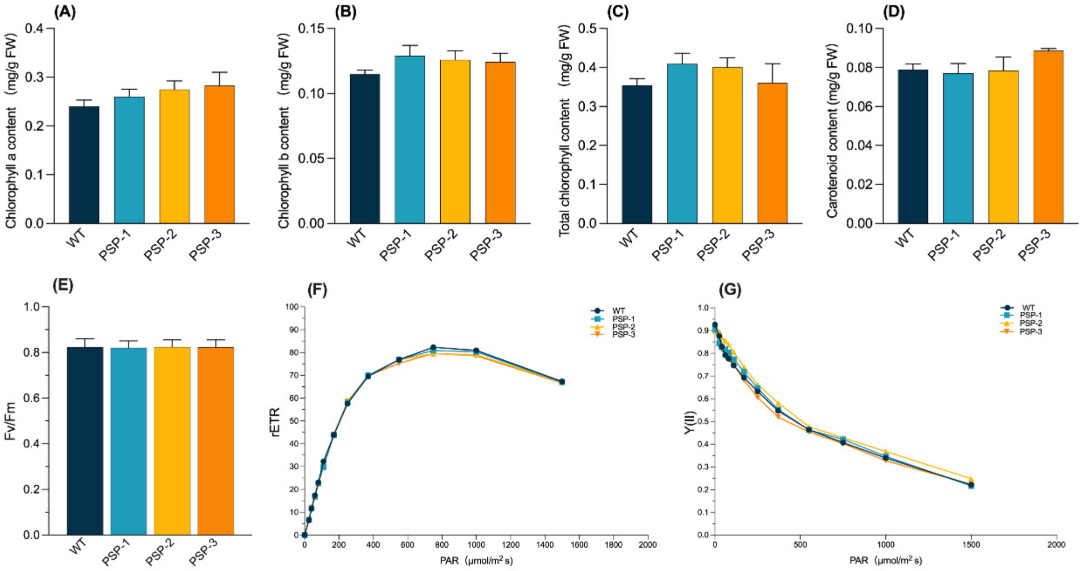

Figure S4. The analysis of photosynthetic pigment and chlorophyll fluorescence-related parameters in duckweed under full nutrition condition (Datko). **(A)** Content of Chlorophyll a; **(B)** Content of Chlorophyll b; **(C)** Total chlorophyll content; **(D)** Carotenoid content. The content of photosynthetic pigment was measured after the samples were cultivated under full nutrition condition for 9 days; **(E)** The value of Fv/Fm; **(F)** The value of rETR; **(G)** The value of Y(II), the chlorophyll fluorescence-related parameters were measured after the samples were cultivated under full nutrition condition for 6 days. Each statistic is the mean ± standard error (n=9, from three independent experiments with three repeats of each, the data no average value in Figure **F** and **G**).

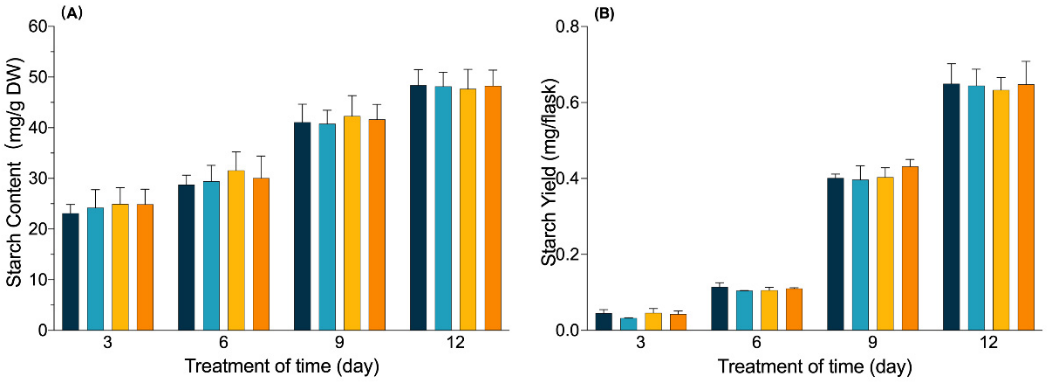

Figure S5. The content of starch , yield and the soluble protein in WT and three *AtPSP1* transgenic lines under full nutrition condition in different treatment of time. **(A)** The starch content; **(B)** The starch yield. Each statistic is the mean ± standard error (n=9, from three independent experiments with three repeats of each).

Table S1 The primer sequences in the study.

Primer name	Primer sequence（5′-3′）	Note	
AtPSP1-F	CCATGGGGATGGAAGCATTAACTACTTCA	The primers for genes amplification	
AtPSP1-R	GGGTTACCCTTAGTCCAATGAGTTTATGAG		
AtPSP1-RT-F	GAAAGGCTGTTGCTGAAT	The primers of genes for RT-PCR	
AtPSP1-RT-R	TCGTTCTCATCAAATCCC		
Lt18S-RT-F	ATACCGTCCTAGTCTCAACCA		
Lt18S-RT-R	ACAAATCGCTCCACCAAC		
AtPSP1-qRT-F	GGTTGTGCCAGTTCAGGT	The primer of *AtPSP1*	The primers of genes for qRT-PCR
AtPSP1-qRT-R	ACGAAGCAATTTGGGATG		
Lt18S-qRT-F	ATAAACGATGCCGACCAG	The primer of *Lt18S*	
Lt18S-qRT-R	TCAGCCTTGCGACCATAC		
LtAPK1-qRT-F	GAAGGAGCCAGTGGTGAA	The primer of sulfur assimilation related genes	
LtAPK1-qRT-R	AGTCGATACAGGGCGAAG		
LtAPR1-qRT-F	TCGCCATAGCTTTCAGTG		
LtAPR1-qRT-R	TTTCAGGGTTCAATCTTCC		
LtPAPSS1-qRT-F	ATGTCTGTTCCCATCGTTC		
LtPAPSS1-qRT-R	TCTTCTTTGTGGTGCTTGT		
LtSERAT1-qRT-F	GCGGCTAATCAAGTGCTG		
LtSERAT1-qRT-R	TTTCGGCGTAACTCCTCA		
LtSIR1-qRT-F	GTGAGCGTCCAGGATTTA		
LtSIR1-qRT-R	CCGATGCGATTAGTGAAG		
LtSULTR1-qRT-F	TTTCTTCGGAGTCATCTT		
LtSULTR1-qRT-R	CATTACACCAGGAATCTTT		
LtSULTR2-qRT-F	AGCTCTGGCTGCAAGACT		
LtSULTR2-qRT-R	ATGGCTGTAGGAGTGAAGTA		
LtSULTR3-qRT-F	TCGCCAACGCCTGCTACT		
LtSULTR3-qRT-R	CGCCCAAGTCCAAGACCACA		
LtSULTR4-qRT-F	TGGGTCTGGTGGACTATGA		
LtSULTR4-qRT-R	ACAAATGCAAGGGAGGAG		
LtSUOX1-qRT-F	GGCGTATGAAATGAATGG		
LtSUOX1-qRT-R	GTCAACTATGGGTGGGAAC		
LtAPS1-qRT-F	ATAGAGCATAGCGTCGTTG	The primer of starch metabolism related genes	
LtAPS1-qRT-R	TTCATCCTCCGTCTCATAG		
LtSSS1-qRT-F	TGTGGCTCTTTGCCTGTA		
LtSSS1-qRT-R	TTCAACTTCGTGCTCTGC		
LtGBSS1-qRT-F	CGGGTAACAGGAAGGGTG		
LtGBSS1-qRT-R	TCCGGTTCTTTGGTGATTGA		
LtAPL1-qRT-F	CACCGTATCCCAAGTCCC		
LtAPL1-qRT-R	TCACCGTCTCGAAATCGT		

LtISA1-qRT-F	GCACGCCTCTGAGCAACCCT	The primer of starch metabolism related genes	The primers of genes for qRT-PCR
LtISA1-qRT-R	TGCCCATTCCATTCTGACCACA		
Ltα-Amy1-qRT-F	ATAATCCGCTCAGAGTTCG		
Ltα-Amy1-qRT-R	ACCGATACCTTACCCTCC		
Ltβ-Amy1-qRT-F	GGCATTCATTGGTGGTAC		
Ltβ-Amy1-qRT-R	GTCGTGCTTCTTGAGGGT		
LtPFK1-qRT-F	TCGGAATAATCGCTGTCTC	The primer of carbon metabolism related genes	
LtPFK1-qRT-R	CGTGAACTTGTGCCTTACC		
LtFBA1-qRT-F	AACCTCAATGCCATGAACC		
LtFBA1-qRT-R	TCCACCTTACCTGCCCAC		
LtPK1-qRT-F	TCAGGCAGAGCAGCAAAG		
LtPK1-qRT-R	CTGGGTTCCCGAGAAAGT		
LtPDC1-qRT-F	GCCCAATCTTCAACGACTA		
LtPDC1-qRT-R	CTTCATCAGAACGCACCC		
LtACO1-qRT-F	GGAGTGACCGCAACAGAT		
LtACO1-qRT-R	GCACCGTATTCAGGAGACA		
LtIDH1-qRT-F	AACGACATACGGCTTCTA		
LtIDH1-qRT-R	TTACCCATTACCTGACAAC		
Lt2OG-DH1-qRT-F	ATTCACGGAGATGGGAGTT		
Lt2OG-DH1-qRT-R	CAGCGGTCGGATCAGTAG		
LtSDH1-qRT-F	AATGTAACCAAGGAGCCG		
LtSDH1-qRT-R	TTTGCGCCATGAACTGAT		
LtFUM1-qRT-F	AACGACATACGGCTTCTA		
LtFUM1-qRT-R	ATTACCCATTACCTGACAAC		
LtMDH1-qRT-F	GCTTCATCTGGGTCTGGT		
LtMDH1-qRT-R	CGTTCGTCATCGTCGTTA		
LtCS1-qRT-F	TTGGACTATGGCGGAAAT		
LtCS1-qRT-R	GCAGTATGGGCGCTAACA		

The sequence of genes

AtPSP1

ATGGAAGCATTAACTACTTCAAGGGTTGTGCCAGTTCAGGTCCCCTGTAGAAAGC
TCTCTTCACTTTTTGCTAATTTTTCTTGCCTTGAGTTAAGGAGATATCCTTGTAGAGG
TCTTGTCTCCATTATGAACCATCCCAAATTGCTTCGTCCAGTGACTGCTTCTGTGCA
GCCTCACGAGTTGTCGACTTTGGGGCATGAAGGCAACATTGTTCCTTCTAAAGAA
ATTCTTGATCTTTGGAGAAGTGTTGAAGCGGTATGTTTCGATGTGGACAGCACGGT
TTGTGTGGATGAAGGTATTGATGAACTTGCTGAGTTTTGTGGAGCTGGAAAGGCT
GTTGCTGAATGGACTGCCAGAGCTATGGGTGGATCTGTTCCATTTGAAGAAGCTCT
AGCTGCAAGACTTTCTTTATTCAAGCCTTCTCTTTCAAAAGTAGAGGAATATCTCG
ACAAGAGACCCCCAAGGCTATCTCCTGGCATTGAAGAGTTGGTCAAGAAACTCA
GAGCCAATAACATTGATGTCTATTTGATATCTGGAGGTTTTCGACAGATGATCAAT

CCTGTAGCATCGATTCTTGGCATCCCTCGGGAGAATATCTTTGCCAACAATCTTCTA
TTTGGAAACTCCGGCGAGTTTCTGGGATTTGATGAGAACGAACCTACGTCAAGAA
GTGGAGGCAAAGCCAAGGCAGTGCAACAAATACGAAAGGGTCGTCTCTACAAG
ACTATGGCCATGATTGGAGATGGTGCTACTGATCTCGAAGCACGTAAACCTGGTG
GCGCAGATTTGTTCATATGCTACGCTGGAGTTCAGCTTCGTGAAGCCGTTGCAGCA
AATGCAGACTGGCTTATCTTTAAGTTTGAATCTCTCATAAACTCATTGGACTAA

Lt18S

CTCAAAGATTAAGCCATGCATGTGTAAGTATGAACTAATTCAGACTGTGAAACT
GCGAATGGCTCATTAAATCAGTTATAGTTTGTTTGATGGTACCTGCTACTCGGATA
ACCGTAGTAATTCTAGAGCTAATACGTGCACCGAACCCCGACTCCCGGAAGGGG
CGCATTTATTAGATAAAAGGTCGACGCGGGCTCGTCCCGCAGATTCGATGATTCA
TGATAACTCGACGGATCGCACGGCCTTCGCGCCGGCGACGCATCATTCAAATTTC
TGCCCTATCAACTTTCGATGGTAGGATAGGGGCCTACCATGGTGGTGACGGGTGA
CGGAGAATTAGGGTTCGATTCCGGAGAGGGAGCCTGAGAAACGGCTACCACATC
CAAGGAAGGCAGCAGGCGCGCAAATTACCCAATCCTGACACGGGGAGGTAGTG
ACAATAAATAACAATACCGGGCTCTTCGAGTCTGGTAATTGGAATGAGTACAAT
CTAAATCCCTTAACGAGGATCCATTGGAGGGCAAGTCTGGTGCCAGCAGCCGCG
GTAATTCCAGCTCCAATAGCGTATATTTAAGTTGTTGCAGTTAAAAAGCTCGTAG
TCGGACCTCGGGTCGGGTCGGGCGGTCCGCCTCTGGGTGTGCACCGGCCGTCCCG
TCCCTCCTGCCGGCGATGCGTTCCCGCCCTTGATTGGGCGGGTCGTGCCACCGGC
GCCGTTACTTTGAAGAAATTAGAGTGCTCAAAGCAAGCCCACGCTCTGCATACGT
TAGCATGGGATAACACCACAGGATTCCGGTCCTATTGTGTTGGCCTTCGGGGTCG
GAGTAATGATTAAGAGGGACAGTCGGGGGCATTCGTATTCCGTAGTCAGAGGTG
AAATTCTTGGATTTACGGAAGACGAACGACTGCGAAAGCATTTGCCAAGGATGT
TTTCATTGATCAAGAACGAAAGTTGGGGGCTCGAAGACGATCAGATACCGTCCT
AGTCTCAACCATAAACGATGCCGACCAGGGATCGGCGGATGTTGCTCTAAGGAC
TCCGCCGGCACCTCATGAGAAATCAGAGTCTTTGGGTTCCGGGGGGAGTATGGTC
GCAAGGCTGAAACTTAAAGGAATTGACGGAAGGGCACCACCAGGAGTGGAGCC
TGCGGCTTAATTTGACTCAACACGGGGAAACTTACCAGGTCCGGACATAGCAAG
GATTGACAGACTGAGAGCTCTTTCTTGATTCTATGGGTGGTGGTGCATGGCCGTTC
TTAGTTGGTGGAGCGATTTGTCTGGTTAATTCCGTTAACGAACGAGACCTCGGCC
TGCTAACTAGCCACGCGGAGGCGACCCTCCGCGGCCGGCTTCTTAGAGGGACTA
CGGCCGTTCAGGCCGCGGAAGTTTGAGGCAATAACAGGTCTGTGATGCCCTTAG
ATGTTCTGGGCCGCACGCGCGCTACACTGATGTATTCAACGAGTTTATAACCTCG
GCCGGAGGGCCCGGGTAATCTTTGAAATTTCATCGTGATGGGGATAGATCGTTGC
AATTATCGATCTTCAACGAGGAATTCCTAGTAAGCGCGAGTCATCAGCTCGCGTT
GACTACGTCCCTGCCCTTTGTACACACCGCCCGTCGCTCCTACCGATTGAATGGT
CCGGTGAAGCGCTCGGATCGCGGCGACGAGGGCGGTCCCCCGCCCGCGACGTCG
CGAGAAGTCCGTTGAACCTTATCATTTAGAGGAAGGAGAAGTCGTAACAAGGTT
TCCGTAGGTGAA

LtAPK1

ATGTCGGATGCTCTGGGATCGGCTCTCGGAGGACGCTTATGGTGCTCTGCGCCCG

AAGAAAGATCTGGATTCTTCGGCCGTGGCGATCTGAGGAACTTCTCCGGGATCAG
GATTTTGAATAGGAGAGTGAAGGAGCCAGTGGTGAAATCGGAAGCATTCCGGCA
GATCATGGCCGGAGAGAGATCCAGAAACCGGAAGGATGTTGATGCTGCATTGGC
TCCCGTAGTTGAGACGAAACTTCGCCCTGTATCGACTGTCGGGAGATCAACGAAC
ATTGCCTGGCATGAATGTCCCGTTGGAAGATTCGATCGAGAGAAATTGCTAGGGC
AGAAAGGATGCGTCATTTGGATCACAGGTCTCAGTGGCTCAGGGAAAAGTACGC
TGGCATGCGCTTTGAGCGGGGAGCTTTATTCCAGAGGATGTTTATCTTATGTTCTTG
ATGGAGATAACGTCAGACATGGACTGAACAAAGATCTTAGCTTCAAACCAGAAG
ATCGCGCGGAAAATATTCGAAGAATCGGAGAAGTGGCAAAGCTCTTTGCAGATG
CCGGTATCATCTGCATTGCTAGCTTAATTTCCCCTTATAGAAAGGATCGAGATGCTT
GCCGCGCTCTTCTTCCCGATCCTACATTCATTGAGGTTTTCTTGGACATACCGCTGG
AAGTATGTGAAGCTAGGGATGTCAAAGGCCTGTACAAGCTTGCACGAGCAGGCA
AGATTAAAGGATTTACTGGGATAGATGACCCATATGAGCGGCCCTTCAATTGTGAG
ATTTTAATACAGCAGAAGGATGGGGTTTGCCCCTCCCCGGGAGACATGGCAGAAC
AAGTCGTTCTCTACCTGGAAGCGAATGGCTTTCTAGATGTATAG

LtAPR1

ATGGCGACGATGGCTGCTTCTCTCTCTTCAACCCGTTCCTTCTCTCTACGCCAGCT
GAAGGCATCCAGGACTATATCAGGTATGCCTGCAGAATTTCCCGCGGCGATCATC
GGCCAAAGGGAGCCACTCGTTCGACTGCAAGTACAATGCATGAAGCCAATGAGA
GGGAGATCCCCTAGAGCTTCTCTCTCCTCATCAGTCACCGGAGAGACCGCTGAAT
CTCAAGGAAAGGCGGCGGCCAAATTGAAGGAAGCCAATTTCGAGAAACTGGCG
GAAGACCTGGACGCGGCCTCTCCCCTGGCGATCATCGACAAGGCTCTGGACTTGT
TTGGCAACGATATCGCCATAGCTTTCAGTGGCGCGGAGGATGTGGCTCTAATTGAG
TATGCCCGGTTAACAGGGAGGCCTTTCCGAGTATTCAGCCTTGACACAGGAAGAT
TGAACCCTGAAACATACCACTTCTTTGACGCTGTGGAGAAGCACTACAACATTCA
CATAGAGTATGTTTTCCCGGAGGCAGTAGAAGTTCAAGGACTGGTTAGAAACAAA
GGCCTTTTCTCTTTCTACGAAGATGGTCACCAAGAATGCTGCAGAATAAGGAAGG
TGAGGCCTCTGAGGAAGGCTCTTAAGGGTCTTCGCGCTTGGATTACAGGCCAAAG
GAAGGATCAGTCTCCGGGCACCAGAGCCAGCATACCCGTCGTTCAGGTTGATCCA
TCTTTTGAAGGATTGGACGGTGGAGCAGGAAGTTTGATAAAATGGAACCCGGTGG
CCAACGTTGAAAGCGCACATATTTGGAGTCTTTTAAGAATAATGAATGTTCCTATC
AACTCCCTACATTCCAAGGGATACGTGTCGATTGGTTGTGAGCCTTGTACAAGGG
CAGTACTTCCCGGGCAGCATGAGAGAGAAGGGAGATGGTGGTGGGAAGATGCCA
ATTCAAAGGAGTGTGGTCTCCATGAGGGTAACGTCAAACAAGACTTGGAGAAGC
CGAAGACCGGCAACGGGGCCTCTAATGGTCCCTTGGATCTCTTCCAAACATCCTC
AATTGTGAGCCTCAGCAGGCTTGGGATAGAGAACTTGCTCAGAGTTAAGGGAAG
GAGAGAGCCATGGATTGTTGTGTTGTATGCGCCTTGGTGCGGCTTTTGCCAGGAA
ATGGAGGGTTCTTACTTGGAGCTTGCGCAGAGGCTCTCTCCTTCAGGCATCAAGG
TAGCAAAATTCCGAGCTGATGGTGACCAAAAGAAATTTGCGGATGAGAAATTGC
AGCTGGATAGCTTCCCAACTATCCTTTTCTTCCCCAAACACACTTTAAAGCCTGTC
AAGTATCCTTCCAAGAACAGAGACTTGGACTCTCTTCTAGCATTTGTGAATACCCT
TTACTAA

LtPAPSS1

ATGGTGTCCATGGCAGCGGGTCTCTCCTGGGCTCGCAACCCAGCTGCCCCTTTTCT
CAGAACCCACCACCATCTCTTCCCCAAGATCGGATTTTCCCAGAAGTTCGAGTCC
AAGATCGCTCGCGGCTCACTCCAAGTGCGATGCAGTCTCATAGACCCCGACGGCG
GATCGCTAGTGAACCTGATCGCTCCAGAAGGCCTTAGAGAGACTAAGAGCCGAG
AAGCGCTCTTACCCAGCATGCCCAGAATCAAGCTATCGAATATCGATCTCCAGTG
GGTTCACGTCCTCAGCGAGGGCTGGGCTAGCCCTCTCCGCGGCTTCATGAGAGAA
GCCGAGTTCCTCCAAACACTTCATTTCAACTTCCTCCGTCTTCAAGACGGTACGGT
GGTGAACATGTCTGTTCCCATCGTTCTCGCCATCGACGATTCGCAGAAGCAGGAG
ATCGGCAATGGCAGAAATGTCACTCTGGTCGACGAAAACGATAATCCCGTGGCG
ATCTTGAGCAACATCGAGATATACAAGCACCACAAAGAAGAGAGGATAGCAAGA
ACATGGGGAACAATAGCCAAGGGTCTCCCTTACGTCGAAGAAGCTATCACGACG
GCTGGAAACTGGCTCATCGGAGGCGACTTAGAGGTCTTAGAGCGAATCAAATAC
AATGACGGTCTTGATCAGTACCGATTATCGCCTTCAGAACTCCGCGATGAGTTCAC
GAAAAGGAATGCGGATGCCGTCTTTGCTTTCCAGCTTCGGAATCCTGTCCACAAC
GGGCACGCCTTGCTCATGACTCACACTCGTGAGCGACTTCTTGAGATGGGATACA
AGAACCCAATTTTGCTACTGCATCCGCTGGGTGGATACACTAAAGCAGACGACGT
ACCGCTATGGTGGAGAATGAAACAACACGAGAAGGTGCTGGAGGAAGGGGTTTT
GAATCCAGAGACAACGGTGTTGGCAATATTTCCGTCTCCGATGCTGTATGCAGGG
CCGACTGAGGTTCAATGGCATGCGAAATCGAGGCTGAATGCTGGTGCGAATTTCT
ATATTGTGGGACGGGATCCGGCGGGGATGAGTCATCCGACGGAGAAGAGAGATC
TGTATGATCCTGAGCATGGTAAGAAGGTGCTGGGGATGGCTCCCGGATTGGAGAA
GCTGAATATCATTCCGTTTAGGGTAGCAGCATATGACAAAAAACAAATGAAGATG
TCCTTCTTTGACCCATCAAGGCCCGACGATTTCATCTTCATATCAGGCACCAAGAT
GAGGACCCTCGCAAAAAACATGGAGAGCCCTCCAGACGGGTTCATGTGCCCCGG
CGGCTGGAACGTGCTTGTCGAATACTATCAAGGCTTGCTTTCTTCAGAAGACAGC
GGTCTTCGTCAATCTGTCCCTGCTTAA

LtSERAT1

ATGCAGTTTCCTAGAGAAATCCATAGGAAACCGCGTGTTCTTGAGCCGTATTTCTT
TTCCAAGTCTAGAGAGATGGAGAACGAGATGTTCGGCATCTCAATCCCACTACTA
AGCCAGACCGCCTTGGGAATGGAGGCTTCATCGCCCGGCGTTGAACAAAGCTTG
ACCGTGCCGACAGAGGCGTTGACTACTCACAGTCAACCGTATTATCTGACGGGCA
GCTACCCGTTCTTCTCCACTCTACAGGAGGCAGCCATCAACAGTCTTCATTCTCGG
GATCTATTTCTTTCTGCGCCTGCTCCCTCGATCCCCGGTTCTTTCTCGGCGTCAAAT
ACTTGTACATTTGGAGCTGAAGACGATGCAGAGATTTTAGCGCCAGGAAGATGGA
GCTTGGAGGAACAAATTGGCCAGCAGATGCTTCAAGCCCGATTTTCAAGCGGGTC
TTACGGCGTTCCGGGCAACGAGCTTTCCCTCACGCTCGGATCTCGCCAGCAATCA
GGCATGGAAATGGCTGGAGTTGCGGATCAATGCTCGGAAATGAGCTGCTCCGAG
ATAACTCAAATCTGTTCCCACGAATCAAGAAACCCTGTTCAAATCTCCCAGACGG
TGTTGGCCTCTACTTATCTTCATGCGGCTAATCAAGTGCTGGCGGAAATCACCTCC
CATGCGCTAGAAGATCCGTATAAGCCTCAAGATCCTGATTATGCTTCCGGAAGAG
GGTGTTCGTCGTTGAGTTCTGAGGAGTTACGCCGAAATTCTCCGTATGAACCGAA
GACGTTGAAAACCGAGCTGATCAGTCTGCTGCAGATGATCGACCACGGATACGA

CGAGTGCGTCGACCGAGTGCAGAGCGCCATAGCGACTTTCAAGAACGCGACGAA
CCTAGCTCCCGCCCAAACGCCAGCCCGCTTCGCTCTCCACACGGTCTCCGCTCTC
TACAAAAGCCTGAGGGACAGAATCAGCTGGCAAATGCAGAAATTCAACCGGGA
GCGTGCGTTGGATACGGAGTCGTCTTTAGAGTGCTCGTTCCTCCAGAGGCAGTGG
GAGTTGCAGCAGCTGAAGAGCGAGCACCAGTCGTGGAGGCCTCAAAGGGGGTT
ACCGGAGAAATCAGTCGCCGTTCTTCGGAGCTGGATGTTCCAGAACTTCCTGCAC
CCGTACCCCAAAGACAACGAGAAGCAGCTGCTGGCGATCAAGAGCGGGCTGAC
CAGAAGCCAGGTACTTTTTTCAGTTTTCCTCTCACCAAAAAAAAGACTGACCGCC
ATTAATGTTTTCTGCGAGCTTCAGGTCTCAAACTGGTTCATCAACGCTCGCGTGCG
CCTGTGGAAACCCATGATCGAAGAAATGTATCAAGAGATGGGTAGAAGCTCACG
AGGGGAGGAAGATCCAAGGAACCCAGAATAA

LtSIR1

ATGAAGAAAAAAACGTTGAGGGAGATTATCGAAAAGTATAATCTAAGTGTGATGA
TTACTCCGAACCAGAATCTGATTCTCTGTGACATTCACCGGTCTTGGAGACAGCC
AATCACCACTGCTCGAGCACAGGGAGGATTACTGCTTCCACAGTACGTTGACCCT
CTCAGCATCACTGCAATGGCGTGCCCGGCTCTTCCGCTGTGCCCTCTGGCAATCAC
AGAGGCTGAGAGAGGAATACCAGACATTTTAAAACGAGTTAGAACTGTTTTTGAC
AAGGTGGGCCTCGAGTTCAAGGAATCGGTCGTCATAAGGGTCACCGGATGCCCTA
ATGGATGTGCCAGGCCGTACATGGCCGAATTAGGGCTTGTTGGTGATGGACCTAAT
AGCTTCCAGATTTGGCTGGGGGGGACGCCGAATCAGACAGAATTAGCAAGGGCC
TTCTTGAATAAGGTGAGCGTCCAGGATTTAGAGAAGGTGCTTGAACCATTATTCTA
CCACTGGAAGGGGAGACGTTTACAGGGAGAATCCTTCGGCAACTTCACTAATCGC
ATCGGTTTCGAAGAGCTTCAGAATATAGTGGAGAATTGGGAGGGTCCAACGGATA
GTCCTTCGGAATCAATCTGA

LtSULTR1

ATGGCTGGATGCAAGACGGCGGTATCCAACATCATCATGTCTCTTGTCGTGTTGTT
GACTCTTGAAGTCATCACGCCTCTCTTCAAGTACACGCCAAACGCGGTCCTCTCA
TCGATCATAATATCAGCAGTTATTGGTCTCGTTGACTTTGAGGCCGTTTTCCTTATTT
GGAAGGTCGATAAGTTTGACTTTATAGCTTGCATGGGAGCTTTCTTCGGAGTCATC
TTCGCTTCCGTGGAAATTGGTCTTCTAATCTCCGTGGCTCTATCGTTTGGGAAGATT
TTACTCCAGGTGACGAGACCGAGGACCGCTCTTCTAGGAAATCTTCCAGGAACCA
CTATTTATAGAAACGTTGACCAGTACCCCGAGGCGACAAAGATTCCTGGTGTAAT
GATCATTAGGGTTGACTCTGCCATTTATTTTTCCAACTCGAATTACATAAAGGATAG
GATTTTGAGATGGTTGACTGATGAGCAAGAGAAGGCTGACCAGAGTCAACTTCC
AAAGATAGAATCTGTGATAGTGGAGATGTCCCGTGAGTATCATCTGACTCCCAAA
AACTTATTGGTTCAGGAAATTTGA

LtSULTR2

ATGATTTCTACTCTGGTGGTCAAGCTTTCTAGGGCTGACCTTCATGGGATCAAAAT
TATCAAAAAATGTAGAAGCTGGTCTCAATCCAAGCTCAGTCAAACAGCTGCAATTT
GTGGGCTCACTTGTTGGAGAGGCTGTTAAAATAGGTCTTATTTCAGCGATCATCGC
CCTCACGGAAGCAATTGCGGTGGGCCGGTCATTTGCTTCGGTAAATGGTTACCAG

CTTGATGGGAACAAGGAGATGTTGGCCATGGGTTGCATGAATATTGCTGGATCAAT
GTCATCTTGCTATGTAGTAACAGGTTCCTTCTCAAGAACAGCCGTAAACTTCAGCT
CTGGCTGCAAGACTGCAGTATCAAACATCGTCATGGCTCTAACAGTGATCCTATCT
TTGGAGCTGCTCATGAAGTTGCTCTACTTCACTCCTACAGCCATCTTGGCATCCAT
CATCCTTGCCTCTCTACCTTCATTGGTTGATGTTCGAGAAGCCTTCCGGATTTGGAA
AATTGACAAAGTTGACTTTCTAGCTTGTGTCGGAGCTTTCTTTGGCGTGCTCTTCA
AATCTGTGGAGATAGGTCTTCTAATTGCGGTATTTATCTCCTTGCTAAAGATCTTAA
TCGGGTCTCTTTGGGCTCGAATTGATGCACTCGGAAAGATAGAAGGAACAAGCAT
CTTCTGCAACATCAAGCAATTCCCCATGGCAATTAGAACTCCTAAGATGCTGATTC
TTCGAGTCAACTCCCCTTTCATTTGTTTTGCCAACTCTAGTTCAATTCGTCAGAAGA
TCTTGAGTTGGGTGAAGGAAGATAGAGAAATGACAGAGGAGAACCCAGAAGAT
AGAGCTAGAGGGGTAGTCCTTGATATGTCAAATGTGATGAATATTGACACCACCG
GCCTAATTGCCCTTGAAGAACTTCACAAGCAGCTCCATTCAGATGGCTTACAGAT
GGCAGTAGCCAATCCAGGTTGGCGTTTGATCCACAAGATGAGGATGGCGAAGCTC
TCCAGCAAGATAGGAGAAGACATGTTATTCCTCAACGTAGCTGACGCGGTCGAAG
CTTGCCTCGGACTTTGCAAGAACACTGCTCCACGTCAATAA

LtSULTR3

ATGAGTAGGCTTCTCTCTCTCTCTCTCTCTCTCTCTCTCTCTCTCTCATTCCCCTTTC
CCTCCAACGGTACCGTGATACAGAGAAGTCTCCAGTCATCTCTCTCTAGATCTCTT
TCTCCTTGTAAATCGATGGGGAACGGAGATGGACTAGGGTTTCAAGGAGCAGCA
AGGGATGGAGTAGAGTGTTCCCACAGAGTTCCTGTTCCGCAGTCAAGGCCAGTTT
TGGAGACCCTGAAGGTCAATCTCAAGGAAACCTTCTTCCCTGACGACCCTCTGCG
TCATTTCAAGGGGCAAACGACGTCCCGCAGGGTGCTGATGGCTCTGCAATACTTC
CTTCCCATTCTCGAATGGGGTCCACGCTACAACTTGGCCTTCTTCAAAGCTGACAT
CGTCTCTGGTATTACCATCGCTAGCTTAGCCATCCCTCAGGGGATTAGCTATGCCA
AGCTCGCAAACCTCCCCCCAATCCTTGGTCTCTACTCCAGCTTCGTACCGCCTCTG
GTGTACGCCTTAATGGGGAGCTCGAGGGACCTGGCGGTGGGCACCGTGGCCGTG
GCGTCTCTCCTCATCGGATCCATGCTCGGAAAGGAGGTTTCACCGTTTCAAAATCC
CACTCTCTACCTTCATCTGGCCTTTACTGCAACCTTCTTCGCTGGCGTCTTCCAGGC
AGCTCTGGGTTTCCTCAGGCTGGGATTTATCGTAGACTTTCTCTCTCATGCGACCAT
CGTGGGCTTCATGGGCGGCGCAGCCACGGTTGTTTGCCTTCAGCAGCTGAAGGGT
ATCCTCGGACTCCATCACTTCACCACCGCCACAGACCTCATCTCCGTCATGGAGTC
TATCTTCTCCCAGACCAGGCAGTGGAGATGGGAAAGCGCGGTGCTTGGAGTCGTC
TTCTTGTTCTTCCTTCTCCTCACCCGTTTCCTGAGCAAGAAGAACCCAAAGTTCTT
CTGGATATCAGCAGCAGCTCCATTGGCCTCCGTCGTTCTCGGAAGCGTTTTGGTAT
TCTTTACCCGGGCTGAGAACCATGGGGTCCAAGTGGTTGGGAAGCTAACTAAAGG
GCTCAACCCCCCATCCTTCAGCGATCTTACCTTCACCTCGCCCTATGTAGTGATCG
CTTTGAAGACTGGCGTCGTCACCGGCATCATTGCTCTTGCAGAAGGCATAGCAGT
GGGAAGGAGCTTCTCCATGTTTAAGGGGTATCATATTGATGGTAACAAGGAGATG
ATCGCCATCGGCATGATGAACATAGCCGGGTCAGTCACCTCGTGCTACCTCACAA
CTGGGCCATTCTCGAGGTCTGCAGTGAACTACAACGCCGGGTGCAAGACAGCCG
TCTCTAACATAGTCATGGCGATGGCGGTCATGATAACTCTGTTGTTCCTGACGCCA
CTTTTCTATTACACGCCGTTGGTCGTCCTCTCCTCTATTATTATCGCTGCAATGCTAG

GACTTATTGATTACGAGGCCGCGTTTCACCTCTGGAACGTCGACAAGGTCGACTT
CTCCGTCTGCATGGGCGCCTACTTTGGTGTCATCTTCTGCAGCATAGAGATCGGCC
TCATTATCGCGGTGGGTATTTCAATGCTTAGGATTTTACTCTTTGTGTCGAGGCCGA
GGACTACTGTCCTGGGGAACATCCCCAACTCAATGATCTACAGAAGACTGGATCA
GTACTCCGATGCTCAAGTTGTTCCTGGATTGCTCATCCTTCGTATCGATGCTCCCAT
ATACTTCGCCAACGCCTGCTACTTGCGAGAGAGGATCTTGAGATGGATTGATGAG
GATGAAGAACGAAATAGGGTGAAGGACGAAGGGAGCCTACTGTATGTGGTCTTG
GACTTGGGCGCTGTGGGGAGCATCGATACCAGTGGAATAAGCATGCTGAAAGAG
ATGAAGAAAACGATGGAGAATAGAGGGCTCAAGTTAGTGCTGGCCAACCCGGGG
AGTGAAGTGATGAAGAAGCTCGATAACTCCAAGGTCATGGAAGCCATAGGCCAG
GAATGGATATTCCTCTCTGTGAGTGAAGCTGTTGGGGCTTGCCATTTTCTACTCCAC
TCCTGCAAGGCCGGCGAGGCTGCAGGTGGGGCAGCCATTCCCAGCGACCACTAT
GTTTGA

LtSULTR4

ATGGATATCTCTTACGCCTCGGCGAGCAACACGGACATCGCCGGGATGCTCTCCG
GCGGTTCAGGCTCCGGGAGGCAAGTGAAGGTCATCCCTCTGCAACATCCGAACG
TCTCCAACCTCTCAGCCCCGCCGACGGCGACGCTTCTCATCAAGTGGCTGCCAAA
CTTTACGGGGATGTCGTCTCGTGACTGGACGGCGCTCATTTTGCCTTGTTCGCGAT
GGATCCGGTCCTACAGGTGGAGAGAATACCTGCAGGTCGACCTTATGGCCGGCAT
CACCGTCGGCGTCATGCTCGTTCCACAGGCTATGTCATATGCAAAGTTGGCCGGC
CTTCATCCCATTTATGGACTCTACTCTGGTTTCGTACCTATATTTATTTATGCGATTTT
TGGGTCATCACGGCAACTTGCGATTGGTCCCGTGGCCTTGGTGTCTCTCTTGGTCT
CTAATGTTCTTGGCAACATCGTTGACTCATCGGATCCACTTTACACGGAACTGGCA
ATTTTGCTGGCTTTCATGGTAGGCGTTCTGGAATGCATTATGGGTTTGTTGAGGCTT
GGATGGCTTATACGTTTTATCAGCCACTCTGTTATTTCTGGCTTCACCACTTCCTCA
GCCATTGTTATCGCATTATCTCAAGCCAAATATTTTTTGGGATACAGTATTGTTAGA
AGCAGCAAAATTGTACCTCTTGTTAAGAGTATAATTGCAGGAGCTGGTAATTTTAT
GTGGCAACCTTTTATGATGGGATGCATCATGCTTACAATTCTTCTTGTGATGAAGCA
CCTGGGAAAAACTAGGAAAAAGCTGAAATATTTAAGAGCAGCTGGTCCACTTAC
AGCAGTTATCTTGGGCACAGCTTTCGTGAAAATATTTCGCCCTTCTTCTATTTCAGT
GGTGGGGGATATACCCCAAGGTCTTCCAAAGTTTTCTATTCCGAAAGAATTTGGGC
ATGCAAAATCATTGATATCAACTGCGATGCTGATAACTGGGGTTGCCATATTGGAG
TCTGTTGGCATTGCTAAAGCACTAGCTGTTAGAAATGGGTATGAACTGGATTCAAA
TCAAGAGTTATTTGGTCTTGGTGTGGCAAATATATGCGGGTCATTTTTTTCTGCATA
TCCAACCACAGGATCTTTTTCTAGATCCGCTGTAAACCACGAGAGTGGAGCGAAG
ACTGGATTATCTGGATTGATGATGGGAATTGTAATGGCATGTGCTCTAATGTTTTTG
ACGCCATTATTTACCGATATACCCCAGTGTACATTGGCTGCCATTGTAATATCTGCT
GTAATGGGTCTGGTGGACTATGATGAAGCGATCTTCTTGTGGCGTGTGGACAAGA
AAGACTTTCTTCTATGGACGATTACTAGCATTACAACATTGTTTCTGGGCATCGAG
ATCGGTGTTCTAGTTGGGGTGGGCTCCTCCCTTGCATTTGTCATCCACGAATCAGC
AAACCCCCACATAGGTCTGTTTTCTCGCCATTTCATCCGCATTCTTATTATCATCATC
TATCAATCTCCCATCATGCACCATCACTCACCAATTCATCATTCTTGTAA

LtSUOX1

ATGGCGGCACTGGTGGGTCCTCATGAGTACTCCCAAGAGCCGCAACGCCATCCAT
GTCTTCGGATCAATGCGAAGGAGCCTTTCAATGCCGAGCCGCCGCGCGCTGCTCT
GGTGTCGTCGTACATCACGCCGGTGGATCTGTTCTTCAAGAGGAACCACGGACCT
ATTCCTATCGTCACTGACCTCGACAGATATCGCGTGTCTTTGAGTGGTTTAATCGGC
AAATCGATTCATCTTTCGATGGCAGATATCAGGAATCTTCCCAAGCACACTGTCAC
TGCTACACTCCAATGCGCCGGTAACAGAAGAACTGCCATGAGCAAGAGAAGGAA
AGTGCGAGGCGTCGGCTGGGACGTATGCGCCCTTGGAAACGCAACATGGAGTGG
AGCAAGATTATGTGACGTTCTTGAGCTGGTAGGGATCCCAAGAAATTCATCAAGA
ACAGCTAATGGGGGAAAACACGTGGAGTTCATCAGTGTAGATAGGTGCCCTGAG
GAAAATGGTGGTCCTTACAAGGCGTCGATCCCTCTGAGGCAAGCGACCAATCCA
GACGCAGAAGTGCTGCTGGCGTATGAAATGAATGGCGAGGTCATCAGCCGAGAT
CACGGGTACCCTATTCGTGTGGTCGTCCCCGGTGTAATTGGGGCGCGATCGGTCAA
GTGGCTAGATTCGATCAAGATAATTGACGAAGAATGCCAGGGATTCTTCATGCAG
AAGGATTACAAAATGTTCCCACCCATAGTTGACTGGCACAACATAATTTGGCCCA
CCAGGAAGCCTCAAATGGACTTTCCCGTTCAGTCTGCCATCTGCTCACTGGGGGA
TGAAGACGTTGTGGACAAGGACAAGATAACAGTCACCGGGTATGCCGTCTCTGGT
GGGGGCAGAGGGATTGAACGTGTTGATATTTCAGTTGACGGAGGGAAGACGTGG
ATTGAAGCTAAGAGACAACAGAAGCCAGGTGTCGCCTACGTGGCAGATGGTGTT
GACAGTGACAGGTGGGCGTGGGTCCTTTTCAGCGCTGAAGTCGATGTATCCCCGG
ACATGGAGATTGTTGCTAAAGCTGTCGATTCGGCGGCGAATGTGCAACCAGAAGA
TGTGGGGCCCATATGGAATCTAAGGGGGATCCTGAACACATCATGGCATCGCATT
CGCGTCCGTAGGGTTCCACGGCGCCACGTGGCTGGACTTGGTCATCCTAGATTATG
A

LtAPS1

ATGGCGATGGCCGCGACGAGCATCTCCGGTGTTGCGCCTCGTTTTGGCTCTGCCG
ATTCTACCAGCAATCGACCTGCGAGCGGTGGAGCGACGTTCTTGAGATCTTCGAA
CAGGAATTTGTCTGCCGCATCGTCTTCGCTTGTTGCAGGAGAGTTGCGGATCTCGT
CGTCTGAGTCTCGACGCCGTGGGAATGCGTCCAGCCCCCGGGCGCCGATCGTCGT
TTCACCGAAGGCTGTTTCTGATTCCAAGAGTTCACAGACTTGTCTCGATCCCGAG
GCCAGTAAGAGTGTTTTGGGAATTATCCTGGGTGGTGGAGCTGGAACTCGCCTCT
ACCCATTAACCAAGAAGAGAGCGAAGCCGGCAGTTCCTCTAGGAGCAAACTAC
CGACTGATTGATATTCCAGTCAGCAACTGCTTAAACAGCAACATCTCAAAGATCT
ACGTGCTCACGCAGTTCAACTCTGCTTCTCTTAACCGTCACCTTTCTAGGGCTTAT
GGTAACAACATTGGGGGATACAAGAATGAAGGTTTTGTCGAAGTCCTCGCCGCC
CAACAGAGCCCAGAAAACCCTAACTGGTTTCAGGGAACCGCAGATGCTGTTAGA
CAGTACTTGTGGCTATTTGAGGAGCACAATGTGATGGAATTCCTTATTCTTGCCGG
GGACCACTTATACCGTATGGACTATGAGAGATTCATTCAAGCCCACCGGGAAAC
TGATGCCGATATCACGGTAGCAGCCCTTCCCATGGATGAAAAGCGGGCCACCGC
CTTTGGACTCATGAAGATCGACGAAGAAGGGCGGATAATTGAATTTGCGGAAAA
ACCCAAGGGAGAGCACCTCAAAGCAATGAAGGTCGACACGACAATATTAGGTC
TGGATGATGAGAGAGCAAAGGAGATGCCTTTCATTGCGAGTATGGGTATTTATGT
TGTTAGCAAAGATGTGATGCTTAACCTTCTTCGTAACGAGTTCCCTGGAGCTAAT

GACTTTGGAAGTGAAGTCATTCCTGGAGCCACTTCTGTTGGATTGAGAGTTCAAG
CCTATTTGTTCGACGGGTATTGGGAAGATATCGGTACCATTGAAGCATTCTACAA
CGCGAATCTCGGGATCACGAAGAAACCAGTCCCGGATTTCAGTTTCTATGATCGT
TCGTCTCCGATCTACACCCAACCTCGATATCTTCCGCCGTCCAAGATGCTGGATG
CTGATGTCACTGATAGTGTCATCGGGGAAGGATGCGTGATTAAGAACTGCAAGA
TTCACCATTCGGTCATCGGCCTCCGCTCTTACATCTCCGAAGGCGCAATCATAGA
AGACACTTTGGTGATGGGAGCCGACTACTACGAGACCGACGCGGACAAGAGATT
TCTTGCCGCTAAAGGCAGCGTTCCGATCGGCATAGGCAGGGACACCCACATCAA
GAAGGCGATCATCGATAAGAACGCCAGGATCGGAGAGAACGTGAAGATTGTGA
ATGCAGATAATGTACAGGAAGCAGCAAGAGAGACGGATGGTATTTCATCAAG
AGCGGCATCGTGACTGTTATCAAGGATGCGCTGATCCCAAGCGGAACCATCATC
TGA

LtSSS1
ATGGCGTCTCTAGGGAGAGCAGGACCTCTCTCTTGCTTTCTCGAGCGATCGAGCG
ACAAGCGTTCCTGCATTCGGGGAAGCCGCGGGAAGCTCCAATCCTTCGGATTCTC
CGCGCTCGCTCGCCGCGGATCTAGCACGCTTGATCTGAAGATTCTGAGAGCGCGC
GCCGGAGATGATTACAGAGGTGTAGGCAAGAATTTGCGTACATCCGCGATCGGG
AAGAAAGGATCTCCCGGGGAGGAAGGAGGCGGCGACGGCGATTTGAGCGACGT
CGACGAGGCGCTGAAGGTGACCATCGAGAAGAGCAAGAATGCGCTCGCCGCAC
AGAAGATCCTCCTTCAACAGATCGTGGAGAAGAGAAAAAGCGCGAATCCAGCA
GAAAAAGGCGTCGGCCACAGCGGAAGAAGTTCATCCTCAGCTTCAACCAGCGG
GAGATCCAAGAAGAGCGAGAAGAAACTTTCTTTCTCCTCGAAGGTTGCAGACGA
CGCCCCCGGCGATCAGACTGGAGTGGAGATCGCGGTTGTCCAAGGAACAACCTC
CATCGCCGAATCTCTTGGTCTGCTGAGAGAAGTTGATCTGGAGATCTCCGATTCC
ACGAAAATTTTGGATTCCTTCACCGAGGAAGATGATCCAGAAAAGAACCAAGGA
AGTCTGGAGGAGGATGAAGAACAGAATGCAGATGTTCCTGCGGAACAACAAGA
CGTAGAAGCGCCGCCGCCTCTGGCGGGGGAAAATGTGATGAATATCATCTTGGT
CGCTGCGGAATGTGCTCCGTGGTCCAAAACTGGGGGCCTTGGAGATGTTGCAGG
GGCGTTACCAAAGGCCTTGGCCAGACGAGGCCATAGAGTTATGGTTGTGGTGCC
ACGGTATGGGAACTACTCGGAATCCCAAGACATAGGAGTTCGCAAGTACTATAA
AGTTGACGGCCAGGACATGGAGGTGACCTTCTTCCACGCCTACATCGATGGCGTT
GACTTTGTCTTCCTCAACAGCCCTATCTTTCGCCACCGTGAGAATGCCATCTACGA
GGGGAACCGTGTGGACATTTTGAAGCGCATGGTCCTGCTCTGCAAGGCAGCTGTC
GAGGTGCCGTGGCATGTGCCATGCGGGGGGGTTTGCTATGGGGATGGGAACCTG
GTGTTCATCGCCAATGACTGGCACACGGCGCTGCTCCCTGTCTATCTTAAGGCCT
ACTACCGGGACCACGGCCTCATGAAGTACACTCGCAGTGTTCTTGTCATCCACAA
CATTGCCCATCAGGGACGTGGGCCGATGGAGGACTTCGGGGTCATCGATTTGCCT
GGCCACTACACAGAGATGTTCCGCACTTACGACCCAGTGGGTGGGGAGCACCTG
AACATTTTCGCGGCCGGGCTCAAGTCCGCTGACCGGATTGTCACTGTTAGCCACG
GGTATGCCTGGGAGGTGAAGACGTCTGAGGGCGGTGGGGCCTACACTCTGTGG
TCAACGACAACGACTGGAAGATCTGTGGAATAGTCAATGGCATTGACACTGCTG
ACTGGAACCCGTCCACGGATTCCCATTTAGGATCTGAAGACGGGTATGTAAATTA
CACAGTGGAGACGGTCAAGGAAGGGAAAGCCCAGTGCAAGGCGGCGCTGCAGC

AGGAACTGGGCCTGCCGTCCCGGCCTGAGGTTCCCTTAGTGGGCTTCATCGGGCG
GCTGGACCACCAAAAGGGCGTGGACATTATTGCAGAAGCAATCCCGTGGATGGT
GGCGCAGGACATGCAACTGGTGCTGCTGGGCACAGGCCGGGCCGACCTGGAGG
ACATGCTCCGGGGCTTCGAAGCCCAGTACAGGGATAAGGTGCGCTCGTGGGTCG
GTTTCTCCGTGCGGATGGCCCATCGGATCACGGCAGGGGCGGATGTGCTGCTGAT
GCCATCGCGGTTTGAGCCGTGTGGGCTCAACCAGTTGTACGCGATGGCCTATGGG
ACTGTCCCAGTGGTGCATGCCGTGGGTGGGCTGAGAGACACGGTGCAGCCCTTT
AACCCGTTTGAGGAGACTGGCCTGGGGTGGACCTTCGACAGGGCTGAGTCTGGG
AAGCTGATCCATGCGCTGGGCAACTGCTTCAACACCTTCTGGAATTACAAGCAG
AGCTGGGAGGGGCTGCAGCGTCGGGGCATGACGCAGGACCTCAGCTGGGACCA
TGCTGCTCAGCTCTATGAGGAGGTCCTTGTCGCTGCCAAGTTCCAGTGGTGA

LtGBSS1

ATGGCCTCTGTCACTATTTCAAACGTTGTCTCGGGAAGTACTCATGGTGCGTGTG
ACGTGGAGCAAAGAAAATCGGGTAACAGGAAGGGTGTATCCCTGAGAAGTCAG
ACGAACGCGTTTCATGGACTGAGGTCCCTCAACAAGGTGGACTCGTTTCAATCAC
CAAAGAACCGGAATTCAAGCTTCGCACGAGCTCTTACGAAAGGCGTCCAGCCTG
GGAAATCCAAACATTCTGGCACTGTCGTTTGCTCGGGTGGCAAGTCCATAGTCTT
CGTATCGACTGAAGTTGCCCCATGGAGCAAGACCGGGGGTTTAGGTGACGTTGT
CGGTGGATTGCCTCCTGCCTTGGCGGAACTAGGCCACCGTGTGATGACCGTATCC
CCTCGCTATGATCAATACAAGGACGCATGGGATTCATCTGCCCTGCTAGAGCTTG
AAGTGGCGGGCAAAGTGTACACTGTTCGTTTCTTCCACTCCCACAAAAAGGGCGT
GGATCGCGTCTTTGTTGACCATCCTTTGTTCCTGGAGAAGGTGTGGGGCCAAACC
GGAGGAAAATTGTATGGCCCTGTGGCTGGAGAAGATTACGAAGACAACCAGACT
AGGTTTATACTTCTCTCTCTTGCCGCATTGGAAGCTCCTCGTATTCTAAATTTCAA
ACCTAGCGGATCCTTTTCTGGGCCTTACGGGGAAGATGTTGTCTTTGTATGCAAC
GATTGGCACACTGCAGTCCTACCCTGCTACTTGAAGACCTTGTACAAGTCGCACG
GTATTTACAAAAATTCGAAGGTTGCTTTCTGCATCCACAATATTGCCTACCAGGG
AAGATTTCCCGCCTCCGACTTCTCACAACTCGGTCTCCCGGACAGCTTCAAGAGC
TCATTCGATTTCATTGATGGATATCTAAAGCCTGTGAAGGGGAGGAAAATAAACT
GGATGAAAGCGGGAATCAAAGAATCGGACATGGTTCTGACAGTCAGCCGATTCT
ACGCCGAAGAGCTGATCTCTGGCGTCGCCAAGGGCGTGGAGCTGCACAACGAG
ATTCAAGAGGCGGGCATCCTCGGGATCGAAAACGGGATGGACGTAGACATCTGG
ACCCCAAAAACTGATAAATATATCTCGGCGAATTACGATCCTGCAACGGTGCTC
GAGGCAAAACCCTTGAACAAGGAAGATGTGCAGGCATCCCTCGGGTTGCCAGTG
GACCCCAACGCCCTTCTCTTGGCTTTCATTGGCCGGCTGGAGGAGCAGAAGGGTT
CGGACATTCTTGCCGAAGCCATTCCTAAATTCATCGGCAAGAACATCCAGATGGT
CGTCCTTGGAACGGGGAAGAAGGCGATGGAGAAGCAATTGAAGAAGATAGAGG
AGGCCTATCCAGAGAACGTGAGAGCAGTGGCCAAATTCGACCCTATTCTGGCCC
ACAAACTGTTCGCTGGCGCAGATCTGATGGTTATTCCCAGCAGATTTGAACCCTG
CGGTATTGTTCAACTACAAGCCATGAACTACGGCACGCCGTCTCTGGTTACGAGC
ACCGGCGGACTGGTGAACACGGTGGTGGACGGGTACACAGGCTTCCACATGGGA
GCTTTCAACATCGATTGTGACGCAGTGGACCCACAAGACGTGGAAGCGATCGTG
TCGGCTGTAAACCGGGTCAGTGAAGTTTACGGCACGGAGCAGTTTACGGAGATC

ATTCAGAATTGCATGGCCCAGGACCTCTCCTGGGAGAAGCCCGCTAAGAAGTGG
GAGAAGGCGCTACTGGGCCTCATCTCCGGTGAAGACCAGCCCGAGGAGGCTGCG
GAGCCCACCAAGGAGCTGGCCGCTGCTTGA

LtAPL1

ATGGCGAGAAGTTTTCTGTTCGACATATGGCTTCGTCTTCTTCTGCCAGCGACCGG
CATGTTCATCTTGGCCATGGCGGAGGAAAGAGAAATTTCACAAACCCAAGCTAC
GGTCCACCTTCTGGACAAGCTCCTCCAATGGAGGAATTTCTTAGAGAGAGAATCT
CCGGATCCTGGCCGGAGAATGCTCGTTGCGGCGGCGGTCTGCTTCCTCGCCGCCG
CCGTGTCCAGCGCCGGTGGAGTCGGCGGCGGGTCGCTGTTCCTCCCCATACTTAA
CCTTGTCGCCGGAATCGAGCTCAAGACAGCTTCGAAGTTCTCAGCTTTCATGGTC
GCCGGAACCGCCGTTTCCAACGTCATGTACAGCGTCTTCTCCGGCGCCGGCGCCG
GCGGCCAGCATGTGATCGACTACGATATCGCCTTGCTCTCAGAGCCCTGCATGTT
GCTGGGCGTGACCGTCGGAGTCATCTGCAACGTGGCGTTTCCGGAGTGGCTGGTG
ACGCTGCTCTTCGCCGTCGTTCTCTCTCTATCCACCATTAAAACTTGCAGAGCCGG
AGCTCGGAAGTGGAAGGAGGAGTCGGAGGGGATGAGAGGGAAGAAGGACGAG
AGAAGAGAAAGTGTTAGTGAGAGAGTGAACGACTTGGAAAAGCCTCTTCTAGA
GGATGAGAGAGAAAGTCCAAATAAGACGAAGTTGAAGAAGGTGGGAGTTCTGG
TTTTGGTCTGGTTCTGCTTCTTCCTCCTTCAATTCCTCAGACAAGGCAATGGATCA
AAGGGTGTGATTAACATGGAGAGATGTGGAGTTGGGTACTGGCTCATCACCGTA
TCCCAAGTCCCAGTCGCTCTTCTCTTCACTACCTATATGCTCTACGCAAAGGATCA
GGAGCAGAGACGGGGGTACAGCCAGAGAGACGATTTCGAGACGGTGATCGATA
GTCTACCCAAGATCATATTCCCAGTTGCAGCCCTCATGGCCGGGGTTCTCGGTGG
GCTCTTTGGGCTCGGCGGTGGACTCCTGATGAACCCTGTTCTTCTTCAGGTCGGCG
TACCTCCTCAGATGACGGCAGCAACAAGCTGCTTCATGGTCCTGTTTTCTGCGTC
AATGTCGTCGGTGCAGTACTTGATGATGGGGATGAAGGAGATAAGACAAGCTCT
GATCATGGCGGCGGGATGTGGTCTGAGTTCCATGGCTGGGGTTGTTCTTGTGGAG
AAAGCCGTCGTAAAGTACGGCCGAGCTTCGCTCGTCATCTTCTCCCTTGGCACAG
TGATGGTGTTGAGCTCCGTCTCCATCACTGGCTTTGGAATTGTCGACATATGGAA
AGACTATGTCGATGGTAGAAATCTTGGGTTCAAACTTCCTTGTTGA

LtISA1

ATGGATCATGCCCTGGCGATGTCGAGCTTGCGGGCCTTCACCAGCGGCTGCCATA
ATCGCCGAAATTCAATTACTTCGGATTGTCGCATTTGCCTTCGCCCGGGGTTCTGG
AGCGCCCCTTCGAGAAATGAGAAGGTGAATGCGAAACGTTTGACCGGTAACAGG
AGAGCGGAGTCCATGGTGCGTCTTTCTGTCAGAGCCACGGGTGTCACTGAGGTGG
CGGAGAACGTTGGTGTTCAAAGAGGTTCATCTTTCGGACTCGTCGTTGAAAATGG
CTCTCCAGAGCCCCTTGGCGCCACCGTTCGTGGTGGTGGAGTAAATTTCGCCATCT
TTTCAAGCGGAGCGACATCAGCTTCCCTGTGCCTCTTCAGCCTCGCCGACCTGCA
AGCGAATAAAATAACGGCGGCTATACCTCTTGATCCGGTACTGAACAAAACTGGA
CATATATGGCATGTATTTGTGGAAGGAGGGCTCCATGGCATGCTCTATGGGTACAA
ATTTGACGGGGTCTTTTCCCCTCAAGATGGGCATTACTTTGATTCATCCCTTATTTTA
GTGGATCCATATGCTAAGGCAGTTGTAAGTAGAGATGAGTATGGCATCCCAGGAC
CTGGAGGCAACTGCTGGCCTCAAATGGCTGGCTTGATACCCTCTCCTCATGATGA

GTTTGATTGGGAAGGAGATCTACCGTTATCTTACTCTCAAAGAGACCTTGTAATCT
ACGAAATGCATGTCCGTGGGTTTACACGGCATGATTCCAGTGGTACCAAGTATCC
AGGAACATATCTTGGAGTGACAGAGAAACTTGACCATTTAAAGGAGCTTGGGGTC
AACTGTATAGAACTGATGCCTTGCAATGAATTTAATGAACTTGAGTACTTCAGCCA
TAACTCTGCCACAGGAGAGAAAAAATTTAACTTCTGGGGTTATTCGACGGTTAATT
TCTTTTCACCGATGATAAGATATTCATCAGCTGGTTCGGAGAACAGTGGCCTTGAA
GCAATAAATGAATTCAAGTATCTTATCAGAGAAGCCCATAAACGTGGCATAGAGG
TACTCTTGGATGTTGTATTTAATCACACTGCTGAAGGGAATGAGAGAGGCCCCATT
TTGTCATTTCGAGGTCTCGATAACAGCGTTTATTATATGATTGCACCAAAGGGAGA
GTTCTATAACTACTCGGGATGCGGAAACACTTTCAATTGTAATCATCCTGTCGTTCG
ACAGTTCATCGTGGATTGTCTAAGATACTGGGTGATCGAAATGCACGTGGATGGAT
TTCGTTTTGATCTCGCATCAATCATGACCAGAGCCTCCAGCCTTTGGGATGCCATT
AATGTACACGGAAATCCAATTGAAGGCGACAATATAACCACTGGCACGCCTCTGA
GCAACCCTCCATTGATTGAAATGATCGCCGAAGATCCAATTCTCAGTAAAGTGAA
GCTCATTGCGGAAGCATGGGATGCCGGGGGTCTCTACCAAGTAGGAAGCTTTCCT
CACGGGGGAGTGTGGTCAGAATGGAATGGGCAGTACCGAGACGTCGTACGGCAA
TTCATAAAGGGAACAGATGGGTTCGCCGGGGCTTTCGCCAAATGCCTGTGCGGGA
GTCCCGATCTATACCAGGATGGAGGAAGAAAACCATGGCACAGCATCAACTTCGT
CTGCGCGCACGATGGCTTCACTCTGGCGGACTTGGTCAGCTACAACAACAAGAA
CAACTTCTCCAACGGGGAGAACAACAACGACGGCGAGAATCACAACAACAGCT
GGAACTGCGGACAGGAGGGAGAAACGGCTGCGTTGCAGGTGATAAGGTTGAGG
AAGAGACAGATGCGGAACTTCTTCCTTTGCCTGATGGTCTCCCAGGGAGTGCCGA
TGATCCTGATGGGCGACGAGTACGGCCACTCCAAGGGCGGGAACAACAACACCT
ACTGCCACGATAATCACCTCAACTATTTCCGGTGGGATCAGAAGGAGGAATCTTC
GCGGGATTTCTTCAGATTCTGCCGCCTGATGACCCGATTCCGCCGGCAAGTTATAA
GCAGATCCATCCATCTTCCTCGGCTCTTTCCTGTTTATTGA

Ltα-Amy1

ATGGGGGAATTTCTGATATGGAGGTCATCTTCCGCGGCTGGTCATCTCTCCGTTCA
ACAGTCTGGACGGGAGAGTTTCAAGTTCATCTTTCCTCGAGATCCTGGCAAGAGA
GGGAGATTCTCTCGCTTGGGGTACAATCACAAGACATCAGCGCTCTCAGGATCCA
CAGGATGCCTTCATTGCCGGCATAATCCGCTCAGAGTTCGTCCGATCAGAGCTGG
CATCGCTGAGACTCCAATTGCTGAAGCTCCAGATGCCAACAACACGGACGCCCT
GTTCAAGCAGACATTTCCATTGACGCGAACGAAATCTGTGGAGGGTAAGGTATCG
GTGAGGTTGGAGAAAAAAGGCGATAATGCTGAAGGAAATCGATGTTTGACCATT
CGATGCAACCTCGAAGGGAAGTGGATTCTTCATTGGGGTGTGACGTACATTGAAG
AGGCGGCCAGTGAATGGGATCAGCCCCCTCTTGACATGAGACCCCCTGGTTCTAC
TCCCATAAAGGACTATGCTATTGAGACGCCCTTAAAGAAATCCTCTTCTGCGTCAG
AAGGAGAGATACTGTATGAATTGCAGATAGATGTGAAACCTAATCTCTCTGTTTCA
GCCATTCATTTTGTTCTCAAGGATGAGGAAACAGGAGCTTGGTTTCAGTATAGAG
GGAGAGACTTCAAGGTTCCTCTTCTCATGTGGGAAGATGATGAGATCGTCAGTGG
GGAAAAAGGCTTCAGCATTTGGCCAGATGAATCGACTATGAAGGCGAACGAGAA
AGAAAAAACCGAAGCCAAACGAGAAGTGAAGCTTGTGGAGGAGTACATTGAAA
AGTTTCCAATAGTACAAGAGGAGACATTTCAGAATTATCTCACAGTTTCAGTGAG

AAGAAATGACGAATTGGATAAAAATGTGGTGCATTTTGACACCGATATACCAGGC
CACGTAATTATTCACTGGGGAGTCTGCAGAGATGAAAATAGAAACTGGGAAATTC
CAACGCCGCCATATCCAATGGGTTCAAGAGTATTCCGGGGGAAGGCTTTACAAAC
ATTACTTCAGCCTAAGGCAAATGAGAGTGGAAATTGGGGAGATTTTGTCGTAAAT
GATGAATTTGCTTGTCTCCATTTCGTTATAAAGCTAAACGAGTACAATTGGTTGAA
CAATGTGGGGGATGACTTTTATATCCCTCTCATGACTGAGAGCAGTATACAAGCTA
AGTCTATAATGACCAAAGCGCAAGATATCCTAAAACAAGTCATGGATTCTGAATC
TGAGGCTACTTCATCAATGGAAGTAGAAACAACCCAAGAAGTAGAAGCTGTCTC
CTACACTGATGACATCATCAGTGAGATAAGGAGCCTGGTAACAGACATATCTGCC
GAGAAAGGTAACATTACGAAGAGCAAAGAAGCACAGGAAAATATCCTTCAAGA
GATCGAGAAACTGGCAGCTGAAGCCTATGGAATTTTCAGAAGCTCGATTCCATTA
TTCGTCGAGGAGCCTGTTTCTGATGCGGATTTATTTAAGCCATCTGTACAGATATCT
TCGGGAACAGGGACTGGTTTTGAAATACTTCTTCAGGGATTTAACTGGGAAGCTC
ACAGATCTGGAAAATGGTATGGTGAGCTCTATGCGAAAGCTGAGGAGATAGCCGC
AATGGGTTTCACGACAATATGGTTGCCTCCTCCGACGGAATCTGTGTCCCCTGAAG
GGTACATGCCCAAGGATTTATACAACTTGAATTCGAGATATGGAAATACTGAAGA
GTTGAAGAGCCTTGTAAAGAAGTTCCACGAAGTGGGCATTAGGGTTCTTGGTGAT
GTGGTCCTGAACCATCGTTGTGCTCATGCCCAAAATAAAAATGGCGTGTGGAATG
TCTTTGGTGGCCGCTTGGCATGGGATGATCGAGCTATTGTTTCTGATGATCCTCATT
TTCAGGGTAGAGGCAACAAAAGCAGTGGAGATTTCTTTCATGCGGCTCCAAACAT
TGACCACTCACAGGAATTTGTGAGAACGGATATAAAAGAATGGCTCTGCTGGCTG
AGGGAAGAAATTGGATATGACGGCTGGAGATTAGATTTTGTGAGGGGCTTCTGGG
GAGGCTATGTTAAAGACTATCTTGAAGCAAGTGAGCCCTATTTTGCGGTTGGAGA
ATACTGGGATTCTTTGAGTTACACGTATGGTCAGATGGACTATAATCAAGACGCTC
ACAGGCAAAGAATCATTGATTGGATTAATGCCACCGGCGGAAATGCTAGTGCATT
CGATGTGACCACCAAAGGGATTCTTCATGCTGCTCTGGAAAAATGTGAGTATTGG
AGATTATCGGATGAAAAAGGAAAGCCGCCTGGTGTGATGGGATGGTGGCCATCA
CGTGCCGTCACATTCATAGAGAACCATGACACTGGCTCTACTCAGGGACATTGGA
GATTCCCGTCAGGGAAGGAGATGCAGGGGTACGCATACATCCTGACCCATTCCGG
GACGCCGACGGTCTTCTACGACCACATTATCTCCCACTACCAGCAGGAGATCGCC
ACCTTGATATCTCTCAGGAACCGCAACATGATCCACTGCCGCAGCACTGTGAAGA
TAAAGAAGGCGGAGAGGGACGTGTACGCGGCGGAGATCGATGAGCGAGTAGTG
ATGAAAATGGGCCCGGGTTACTACGAGCCCGAGGGCCCACAATCCTGGTCTGTGG
CCGCCGAGGGCCGAGATTACAAGATCTGGGAAGCGGCTTGA

Ltβ-Amy1

ATGGCCACGGAGATGGAGAAATTAGGAGAGACGAGCGATGAGGATGAGGAGGA
CATGGACATGGACGTGAAAGAAGAGGACGATGACGACGACGGCGAGGAGAGTG
ACAAAGGCGCGTACAGCGGGGGAATGATGATCGCAGGCGCGTTGGCGGACGGG
GAATTCGGTAATAGCCTGGGGAACCGATTCCAGCAGCAACAACATCTTCAGATCT
GCATTTCCGACGGAGATCAGCAGCAGAATCAGCAGGCGATGGCGATGGGCGCAG
GCATCAGCGGCAGCGGGATGAGGAGATGCCGTCCGAAAGAGGAAAAAGAGAGG
ACGAAGCTCCGGGAGAGGCATCGGCGGGCAATAACCGCGCGCATTCTCTCAGGC
CTCCGGCGGCAGGGCAACTACAACCTCCGCGTTAGGGCGGATATCAACGAAGTC

ATCGCCGCGCTCGCCAGAGAAGCTGGCTGGACTGTCCTCTCCGATGGCACCACCT
TCCCCGCAAGGGCAAACTCCCAGTTGCTGCAGACGGGTAGGCCTCCGGGAACGC
CACCGGCATCAATGCCGTCATCGTCTCCGGCACAGAACCCGGCGCCATTGCGAGG
AGTCTATGGGGCCGGAGGTCGATCCACCGACTTCCGATCCTGCCAGATAAAGGGT
GTCTATGCCTCCAGCCCAAGTGAGATACCCGGCCGTCTTCGAGCGTCATCGATGAT
AAGAGCCGAGAGCAGAGGAGGAGAGAGAGATGCGGAGGATTCTCCTCATCTTG
GCACCTGCATCGATGGAATCGATGATAAGCAGAGGATGGATGCCTCTTCCAGAAT
GCAAGAGCGAGATTTCGCCGACACGCCTTACGTTCCCGTATACGTGATGCTACCCT
TGGGGGTCATCAACATGAAATGCGAGCTCGTGGATCCGGATACGTTGGCGAAACA
ACTGAGAATACTCAAGTCCGCCAACGTTGATGGCGTGATGGTTGATTGTTGGTGG
GGGATAGTTGAAGCGCATACTCCACAAGAGTATAACTGGCAGGGGTACAAAAGA
CTTTTCCAAATTGTTCGTGACATCAAGCTTAAACTTCAGGTGGTTATGTCGTTTCAT
GAATGTGGGGGCAACGTTGGGGATGATGTTTTTATCCCGCTTCCACGATGGGTGTG
TGAAATTGGGCAAAGCAACCCTGACATTTTCTTCACAGATAGAGGTGGAAGACGT
AACACTGAAAGCCTTACGTGGGGCATCGATAAGGAAAGGGTTTTAAGAGGCCGA
ACTGCTGTTGAGGTTTACTTTGACTTTATGAGAAGCTTCCGTGTGGAGTTTGATGA
GTTCTTTGGAGATGGAGTGATTTCGGAGATTCAAGTGGGATTAGGTCCATGTGGAG
AGCTACGGTATCCATCCTACCCCGCGAGACATGGTTGGAGATACCCTGGCATAGG
AGAATTTCAGTGTTACGACAAGTACATGATGAAAAGTCTGATGAAGGCAGCAGA
AATGAGGGGTCATGTGTTCTGGGCAAAAGGGCCAGATAATGCCGGCTCGTACAAT
TCTTCACCGCATGAAACTGGGTTCTTTCGTGATGGAGGAGACTACGATAGCTACTA
TGGTAGATTCTTCCTCGGATGGTATTCTAGGTTTCTAACAGATCATGCAGATCGTGT
GCTGTCACTGGCTAAATTGGCTTTTGAAGGCAACCACATTGCTGCAAAGGTGTCG
GGCATTCATTGGTGGTACAAAACAGCCAGCCATGCAGCCGAGTTGACGGCCGGTT
TCTACAACCCTTGTAACCGTGATGGATATGCCGTGATTGCCGAAACCCTCAAGAA
GCACGACACGGCTTTAAACTTTACATGTGTTGAATTACGAACCCTAGACCAGCAT
GAGGACTTTCCGGAAGCACTCGCAGATCCGGAGGGCTTGGTCTGGCAGGTGCTG
AATGCAGCATGGGATGCTCGGATACCGGTTGCCAGTGAAAACGCACTTCCATGCT
ACGATCGAGAAGGGTATAATAAGATACTAGAGAACGCGAAACCCTTCGATGATCC
AGATGGAAGGCACTTGTCGGCGTTCACGTACCTTAGACTTACCCCGATTCTCATGG
AGGCACAGAATTTTATTGAATTTGAGCGCTTTGTTAAGAGAATGCACGGTGAAGC
AGTTCTTGACTTCCCCATGTGA

LtPFK1

ATGGCAGCCGAAGCGCAAACAGCCGTCCAGGATGAGAGAAAGAGAGAGAGGG
AAAGAGGGGAACCCTTCAAAAAACTATCCCTTCTACTGAGCATCATCCTCCTAAT
AGTGGGCGCCGTGGGAGCACCTCTCCTCACCCGTATCTACTTCTTGCATGGGGGA
GCTAGGGTTTGGGTCACGGCCCTTGCCCAGTTTGTCGCCTTTCCACTCATTCTACC
CTTCATTTGCGGCTCCTACGCCCACAGAAGCCGGCGCGGAGATACAAGATTTTTC
GTGCTCTCGGCTAAGGTCATGGTGGCGTCCTTAGGCGTCGGAATAATCGCTGTCTC
AGACGCCTGGTTGTACTCCTACGGGCTCATGTCCCTTCCACTTTCTATCTCTACGCT
GCTTATTGCCACGCAAATAGTGTTCACGGCGCTGTTCGCCTTCCTCCTGGTAAGGC
ACAAGTTCACGGCTTTCTCCGTCAACGCTGTTGTCCTCCTTGTAATCGGCACGGTA
CTGCTGGGCGTTCAATCCAACTCCGAGAGGCCTCCCGGAGAGTCAAACAAGAAG

TATTACATAGGGTTCTTCGCCATGATCGCCGCGGCAGTTCTCTACGGATTGATAATG
CCACTTATCCAGTGGGTCTACGTAAGATTCAAGCAGAGGGAGTCCCTCTCCTCTGT
TCTAGAGATGCAGATCCTCATGTCGATCACCGGCAGCGTGTTCTCCTTGATCGCCA
TGGCCATTAACCACGACTTCGCGGCCGTTTCTCGGGAGGCGGAGATATACGAGCT
GGGAAAAGCAAAGTACTACGCGGTTTTGGTGGGCACGGCGATCTTCTGGCAGAT
GATGACTATAGGTTTCTTAGGAGTGATCGCTTGTTCCTCCGCTCTCCTCGCGGGAG
TCGTCGTAGCCATGCTCCTGCCGGTAGGAGAGATCGTGCCGGTTCTGCTTCTGGGA
GAGAAATTCACCAGCGCAAAGGGAGTCGCCACGGCCTTATGTCTATGGGGATTCA
TCTCCTATCTCTACGGTGACTTCCGGCAGACGAAAAAGAAGCCGGCAGCGCCGTC
AGACGACTGCCGCGTTTAG

LtFBA1

ATGTCTGCTTACCGCGGGAAGTATGCCGATGAGCTCATTAAGAATGCGGCCTACAT
TGGAACTCCCGGAAAGGGCATCCTCGCCGCCGACGAATCCACCGGCACAATTGG
CAAGCGCCTATCCAGCATCAATGTCGAGAATATTGAATCAAATCGTCGTGCCCTCC
GTGAGCTCCTCTTCACCACTCCTGGCGCACTCCAGTACATTAGTGGCGTCATCCTC
TTCGAAGAAACCCTTTACCAAAAAACTGCTGCTGGAAAACCCTTCGTCGACCTTC
TAACTGAATCTGGCGTACTCCCTGGCATCAAAGTTGACAAGGGCACCGTAGCCCT
TGCTGGCACCGACGGTGAGACAACCACCCAAGGTCTCGATGACCTTGGCAAGCG
CTGTGCTACCTACTATGCCGCCGGGGCTCGCTTTGCCAAGTGGCGGGCTGTGCTCA
AGATCGGCCCCACCGAACCCTCCCAACTAGCCATTCAGGAGAATGCCAATGGCCT
TGCTCGCTATGCCATCATCTGCCAGGAGAACGGCTTGGTGCCTATTGTAGAGCCCG
AGATTCTTGTTGACGGGACCCACGACATTGCGCGCTGTGCTGCCATCACTGAGAC
TGTCCTTGCTGCTTGCTACAAGGCGCTCAACGACCACCATGTTCTTCTAGAAGGA
ACTCTCTTGAAGCCTAACATGGTAACTCCCGGGTCGGACTCCAAGAAGGTGGCAC
CGGAGGTGATTGCAGAGCACACTGTACGAGCCCTCCAAAGGACCGTCCCTGCAG
CTGTGCCGGCAATCGTGTTCCTCTCCGGCGGGCAGAGTGAGGAAGAGGCGACAC
GGAACCTCAATGCCATGAACCAGCTCAAGGGGAAGAAACCGTGGAGCTTGTCAT
TCTCGTTCGGGAGGGCTCTTCAGCAGAGCACTCTGAAGGCGTGGGCAGGTAAGG
TGGAGAATGTTGAGAAAGCTCAGTCTGCTTTTCTTACTAGGTGCAAGGCAAATTC
GGAGGCTACGCTCGGCACCTATAAGGGGGATGCGGCCGGCGGTGAAGGCGTCTC
CGAGAGCCTTCATGTTAAGGACTACAAGTACTGA

LtPK1

ATGCACATAGCCCAAGTCCACATCAATCTCCCCACATTGACCAATATGGACAAAA
AGGCTATCTCAACATGGGGGTGTCGCAACAATGCCGACATCATTTCACTCTCATAC
ACGCGTCAGGCCGAGGATGTTCGAGAGCTTAGAGCATTTCTTACTGCTCATAATCT
CGCCGGCACTCAGATATTTGCCAAAATCGAAAACGTAGAGGGATTGAGACACTTT
GATGATATTCTCCGAGAGGCAGATGGAATTATTCTTTCTAGGGGTAATCTGGGCAT
TGATCTCCCTCCAGAGAAGGTGTTTTTGTTCCAGAAATCTGCAGTCTACAAGTGCA
ATATGGCAGGGAAGCCTGCAATAATCACTAGGGTAGTAGATAGCATGACAGATAA
CCTTCGTCCTACTCGTGCAGAAGCCACCGATGTGGCAAATGCTGTTCTTGATGGTA
CTGATGGGATTTTGCTCGGAGCAGAGACACTCAGGGGTCTTTACCCAGTAGAAGC
AATAAAAACAGTTCAAAGGATTTGTTTTGAGGCTGAGTCAGTCTACAACCAACCG

ATTCATTTCAAGGCTATAGTGAAGCATATCGGGGACCCAATGTCAGACGAGGAAT
CCGTCGCCTCTTCAGCAGTTGGATCTGCGATCAAAGTCAAGGCATCAGTCATATTG
GTCTTCACCTCCTCAGGCAGAGCAGCAAAGATGATTGCCAAGTATAGACCACCAA
TGCCCATCATGGCCATCGTCATCCCGAAGATCATGACAAACTCTCTTAAATGGACT
TTCTCGGGAACCCAGCAGGCTAGGCAGTGCCTTGCTGTCAGAGGAGTTTTTCCCG
TTCTTGCCGATCTCAACTCGACGAGGACAATCAGTCCCGGCGAAGATTCTGCCCT
GGAGCTCGCCTTGAACCATGCCAAGTCCGTGGGCTTGGTGAGCACCTACGATCGG
GCTGTGGTATTCCAGAAGATCGGAGACTCCTCAGTCGTCAAGATCATCGAAATCC
CATGA

LtPDC1

ATGGATACGCAAATCGGATCGATCGACGGCGTGAAGCCGGCGAAAATCGATGTC
GGCGCGCTCCCGTCGAACAACTGCTACACTCTCAGCCCTTCCTCGACATCTCCTG
CTTCTCCGACGGAAGCAACCCTTGGCCGGCACATCGCTCGCAGGCTCGTCCAGAT
CGGCGTTACGGACGTGTTCACGGTTCCTGGAGACTTCAATCTCACGCTTCTCGATC
ATCTCATCGCTGAACCGGGGCTAAAGAACATTGGCTGCTGCAACGAGCTCAACG
CTGGCTATGCCGCCGACGGATACGCCAGATCTCGAGGTGTTGGGGCTTGCGCTGT
TACCTTCACCGTCGGAGGATTGAGTATCCTCAACGCCATTGCCGGTGCTTACAGCG
AGAACCTTCCCGTGATCTGTATCGTCGGAGGTCCCAATACCAACGATTATGGAAC
CAACAGGATCCTCCATCACACCATCGGTCTCCCCGATTTCAGCCAGGAACTCCGC
TGCTTCCAAACCGTCACCTGCTACCAGGCCGTGGTGAACAATCTGGAAGATGCGC
ACGAGCAGATCGACGCCGCGATCTCTACCGCGCTAAAGGAGAGCAAGCCGGTGT
ACATCAGCGTTAGCTGCAATATCACCGGAATCCCTCATCCCAGCTTCAGCCGAGA
GCCAGTGCCCTTTTCCTTATCTCCTAGATTGAGCAATCCGGCCGGTCTCGAAGCTG
CCGTTCATGCTGCAGCCGAATTCTTGAATAAGGCCGTGAAGCCAGTAATGGTGGG
AGGGCCGAAGCTCCGAGTAGCGAAAGCCGGAGAGGCGTTCGTCGACTTGGCCGA
CGCTTCTGGCTATGCCGTCGCCGTGATGCCGTCAGCGAAGGGACTTGTTCCAGAG
CACCTCCCTAGATTCATTGGAACTTACTGGGGCGCAGTGAGCACCGCTTTCTGCG
CAGAGATCATTGAATCCGCGGACGCTTACCTCTTCGCCGGCCCAATCTTCAACGA
CTACAGCTCAGTCGGATACTCCCTCCTGCTCAAGAAGGAGAAGTCGATCATCGTG
CAGCCTGATAGAGTTGTGATCGGGAACGGCCCAACTTTCGGGTGCGTTCTGATGA
AGGATTTTCTCGTCGCTCTGGCCAAGAGGTTGCACAAGAACTCGACGGCATTTGA
GAATTACCAGAGGATCTTCGTTCCCGGGGGAGTTCCTCTAGAATGCAAGCCAGAC
GAGCCGCTCAGAGTCAATATTATGTTCAAGTACATTCAGGAGATGGTATCTGGGG
AGACGGCGGTGATCGCTGAGACTGGAGACTCCTGGTTCAATTGCCAGAAGCTCA
GATTACCGGAGGGCTGCGGGTATGAATTTCAAATGCAGTACGGATCGATCGGGTG
GTCTGTGGGGGCAACGCTGGGGTACGCGCAGGCGGCGAAGAACAAGAGAGTGA
TCTCGTTCATCGGCGATGGCAGCTTCCAGGTCACGGCTCAGGATGTGTCCACGAT
GCTTCGAGCCCAGCAGAACCCCATCATCTTCCTCATCAACAATGGGGGATACACC
ATCGAGGTGGAGATCCACGACGGCCCCTACAACGTCATCAAGAACTGGAACTAC
ACCGGCCTCGTGGATGCCATCCACAACGGCCATGGCAAGTGCTGGACCACCAAG
GTGAGGTACGAGAAGGAGCTGAAGGAGGCGATAGAGACGGCGACTGAGGTGAA
GAAAGACTGTCTGTGCTTCATAGAGGTGATCGTTCACAAGGACGATACGAGCAA
GGAGCTGCTCGAGTGGGGCTCCAGAGTCTCCGCCGCCAACAGCCGCCCTCCCAA

TCCTCAGTAG

LtACO1

ATGACGCTTTTGTTTGCCGCAGCGGCTCTCGCGAGGTCTTCCACCTCCCGCATCGT
CCCGCCGGTGGCGGCGACTGCGGCGGCGGCCATGGCGGGGCGGACTTCGCCGTC
TTCCTCTCCGCCGCGAGAGGCGCCGTCTGAGCCCCGACGGTCTCCTCCTTCTTCGC
CGTCTCGGCATTTCGGCGACTGGAGGTCTCCTCTGAGTCACCGCGCTCTGATCCGC
TCTTCCGCCGTCGTGACTGAACAATTCGAGAGGCGATTCGCCACCATAGCTACAA
AAAATTCGTACGAGAGCATCTTGACCTCGCTGTCGAAGCCAGGTGGAGGCGGCG
AGTTCGGGAAGTACTACAGCCTGCCGGCTCTTAACGATCCTAGAATCGATAAACT
TCCATACTCGATACGGATACTATTGGAATCGGCGATCAGAAACTGTGACGAATTTC
AGGTTAAAAGCAAAGATGTGGAGAAGATCATCGATTGGGAGAACACAGCTCCCA
AGCAAGTCGAGGTCCCCTTCAAGCCAGCACGTGTCCTCCTTCAGGACTTCACGGG
TGTGCCGGCTGTTGTTGATCTTGCCTGCATGCGGGATGCCATGAACAACCTTGGAA
GCGATTCAAATAAGATAAATCCTCTGGTTCCTGTTGACCTTGTAATTGACCATTCA
GTTCAAGTCGATGTGGCAAGATCAGAAAATGCAGTCCAGGCGAACATGGAACTT
GAATTCAAACGAAATAGGGAGAGGTTTGGTTTCCTCAAGTGGGGATCCAAAGCAT
TCAAAAACATGCTCGTGGTCCCTCCAGGATCAGGCATTGTCCACCAGGTGAATTT
GGAGTACCTAGGGAGAGTGGTGTTCAACAACGACGGTCTTCTTTACCCTGATAGT
GTTGTTGGTACCGATTCACATACAACGATGATAGATGGTTTGGGAGTCGCCGGGTG
GGGTGTTGGTGGCATCGAAGCAGAAGCTGCAATGCTTGGGCAGCCAATGAGTATG
GTGCTGCCTGGAGTGGTTGGATTCAAGCTATCAGGGAAACTGAATAACGGAGTGA
CCGCAACAGATTTAGTTCTAACAGTGACCCAGATGCTTCGTAAGCACGGCGTCGT
TGGGAAGTTCGTAGAATTCTATGGCCCAGGAATGAGTGAGCTTGCGCTTGCGGAT
CGTGCAACTATTGCCAACATGTCTCCTGAATACGGTGCAACCATGGGGTTCTTCCC
TGTGGATCACGTAACTCTCCAGTACTTGAAATTAACAGGGAGAAGCGATGAAACT
GTGGCTATGATAGAGTCTTATCTTCGGGCAAACAATATGTTTGTGGATTATAGCCA
GTCTCAAATCGAAAGAGTTTACTCCTCTTATCTTGAGTTGAATCTTGAGGATGTGG
AGCCTTGTGTTTCAGGACCTAAGAGACCGCATGATCGGGTTACGTTGAAGGAAAT
GAAGTCAGATTGGCACTCTTGCTTGGACAACAAAGTTGGATTCAAGGGATTCGCT
GTTCCAAAGGAATCTCAGAGTAAAGTCATAGACTTCTCATTCCATGGGACCCCAG
CTCAAATCAAACATGGCGATGTGGTCATAGCTGCAATCACCAGCTGCACGAACAC
ATCAAACCCTAGTGTGATGCTTGGTGCTGCCCTAGTTGCAAAGAAAGCCTGTGAA
CTGGGTCTCGAGGTGAAACCATGGATAAAGACTAGTCTTGCTCCAGGTTCCGGAG
TTGCAACCAAGTACCTGGAAAAGAGTGGATTACAGAAGTACCTAAACCAGCTTG
GATTCAATATCGTGGGTTACGGATGCACGACCTGCATTGGAAACTCGGGTGACATT
GATGAGTCTGTAGCCGCCGCAATTTCTGAAAACGATGTCGTTGCTGCCGCTGTGCT
TTCCGGAAATAGAAATTTCGAGGGGCGTGTTCATCCTCTGACAAGAGCTAATTAC
CTTGCCTCTCCTCCCCTTGTTGTGGCATATGCTCTTGCCGGAACAGTCAACATTGA
CTTTGAGACCGAACCCATCGGAACAGGAAAAGATGGGAAGAAAGTGTACTTCCG
GGACATCTGGCCTTCAACCGAGGAGATCGCAAAGGTGGTGGAATCTAGCGTTCTC
CCAGAGATGTTCAAGGAAACGTACAAGGCAATAACAGAAGGAAACCCAATGTG
GAACGAGCTCTCTGTACCAAGCAGCAAACTCTACTCGTGGGACGAAAAGTCAAC
GTACATCCACGAGCCCCCATATTTCAAGGGTATGACCATGGACCCACCTGGGCCC

CACAGCGTGAAGAACGCCTACTGTTTACTTAACTTTGGAGACAGCATCACCACCG
ACCACATCTCTCCCGCTGGAAACATCCAGAAAGACAGCCCCGCCGCTAAGTATCT
TCTAGAGAGACAAGTGGAGAGGAGAGATTTTAACTCGTATGGCAGCCGACGTGG
CAACGACGAGATCATGGCCAGGGGTACTTTTGCAAATATCCGACTGGTGAATAAG
CTCTTGAAGGGGGAGGTTGGGCCCCACACTGTCCATATCCCTTCTGGGGAGAAAC
TCTCTGTGTTTGATGCCGCCATGAGATATAAGAGCGAGGGGCAGGACACAATTAT
CCTGGCGGGGGCTGAGTATGGAAGCGGCAGCTCCCGGGATTGGGCTGCCAAGGG
ACCTATGCTGCTGGGAGTGAAGGCGGTGATAGCAAAGAGTTTTGAGAGGATACA
CAGAAGCAATCTGGTGGGGATGGGGATAATTCCCCTGTGCTTCAAGACCGGGGA
GGATGCCGAGACTCTGGGGTTGACCGGGGAGGAGAGATTCAACATCGGGCTGCC
TAGTAGCGTGGCGGAGATCAGGCCTGGTCAGGATATTACCGTAACTACTGATACA
GGGAAGTCCTTCACTTGCACCGTCCGATTCGATACCGAGGTGGAGCTAGCCTACT
TCAACAATGGCGGCATTCTCCCCTACGTGATCAGAAACCTGATCAAAGCCGACGG
CTAA

LtIDH1
ATGAGTTGGCTGTCCCTTAGCCACTCATCATACCAGACACTTCTGTTATCATACCC
ACTCATCATACGAGTTCTCATCTCTGTTCTTGGATCTGCTGCCGGCTGCTGTCTCTC
AGATCTGCTCGCCATGGCTTTCGAGAAGATCAAGGTCGCCAATCCGATCGTTGAA
ATGGACGGCGACGAGATGACTCGCGTTATCTGGGAGTTTATCAAGAGCAAGCTGA
TTTTCCCCTTCTTGGATTTGGACATCAAGTACTTTGATCTTGCACTTTCTTACCGTG
ATGCCACAGACGACAAAGTCACTGTTGAAAGCGCAAAAGCAACACTCAAATAC
AATGTTGCCATCAAGTGCGCCACAATAACACCAGATGAGGCTCGTGTGAAGGAG
TTCAACTTGAAATCTATGTGGAAGTGTCCTAATGCCACAATCCGGAACATCTTGAA
TGGCACGGTGTTTAGAGAACCCATCATCTGCAAGAACGTTCCTGGCCTTGTTCCTG
GATGGAGTAAGCCCATATGCATCGGAAGGCATGCCTTTGGAGATCAGTACCGGGC
TACTGATGCAGTCATCAAAGGACCTGGAAAACTGAAACTCGTATTTGAGGGCAA
GGAGGAAGAGATAGAACTCCAAGTTTTCAACTTCACTGGGGAAGGAGGAGTAGC
TCTGGCAATGTACAATACTGACGAGTCTATTCGCTCATTTGCCGAGGCATCAATGG
CCACTGCTTATGCCAAGAGGTGGCCTCTTTATTTAAGCACCAAGAACACAATTCTG
AAGAAATACGACGGAAGGTTTAAGGACATATTCCAGGAGGTGTACGAGGCAGAG
TGGAAGTCAAAGTTTGAAGCTGTGGGGATATGGTCGGTCAACTTATCTGCCACGT
CATCAGAGTTCTATGAAATGAAGATTTTATTATTATTTTTTTATTTTTTATGA

Lt2OG-DH1
ATGGCGTGGTTCAGAGCAGTGTCAGGGATGGCGAGGCATGCCGTGCGGAGGAAC
CTCGTTCAGGGATGTCACCATGTAGCTAGGGCTCACCGTTGTCCTCTACGCTCCCG
CGGCTTCCGTTCGACTTCTCTGAGGCCCAACGCCGCTCCGATTCCTCGTCCCGTGC
CGCTTTCCAAGCTCACGGATAACTTCCTAGACGGCACGAGCAGCGTTTACCTCGA
GGAATTGCAGAGAGCGTGGGAAGCCGACCCCGACAGCGTCGACGAATCATGGG
ACAACTTCTTCAGGAACTTCGTCGGCCACGCCGCTACATCGCCAGGAATCTCCGG
CCAGACAATCCAGGAGAGCATGCGGCTGCTACTTCTTGTCAGAGCATACCAGGTA
AACGGCCACATGATTTCCCAATTAGATCCTCTTGGGCTTCAGGAGCGCGAAATTC
CAGAAGACCTCGATCCAGCCTTCTACGGATTCACTGAAGAAGATCTAGACAGAG

AATTCTTCATCGGAGTATGGAGAATGTCTGGGTTCCTCTCCGAAAATCGCCCTGTT
CAAACTCTTCGATCCATTCTAGATCGCCTCAAACAAGCCTACTGCGGCAGCGTTG
GATACGAATACATGCACATCGCCGACAGAGATCAATGCAACTGGTTGCGAGACAT
GATCGAAACTGTTAAACCTCGAGCCTACGGGAAGGATCGACAGGAAGTCATCCT
AGATCGACTCATCTGGAGCACCCAATTCGAGAATTTCCTCGCTACAAAATGGGTA
ACAGCGAAGAGATTCGGCCTCGAAGGAGCGGAGACTCTGATTCCAGGGATGAAG
GAGATGTTCGATCGAGCTTCAGATCTCGGAGTAGAGAGCATCGTCGTTGGAATGC
CTCACAGAGGTAGACTGAATGTTCTTGGGAACGTCTTCCGCAAACCACTTCGCCA
AATTTTCAGCGAATTCAGCGGTACCGGCTCAAAAGCCATGGAAGAAGTTGGACTC
TATACTGGAACCGGCGACGTGAAGTATCATCTGGGTACCTCCTATGATCGACCCAC
TAGAGGAGGAAAGAGAATTCATCTCTCTCTGGTGGCAAACCCTAGTCATCTTGAA
GCTGTTGATCCAGTTGTTCTTGGGAAAACTAGGGCAAAACAGTATTACTCCAATG
ACCTCGATCGGACCAAGAATTTGGCAGTTTTGATTCACGGAGATGGGAGTTTTGC
CGGGCAGGGAGTGGTTTACGAGACTCTGCATCTCAGTGCTCTTCCCAACTACACC
ACCGGTGGTACTATTCACATTGTGGTGAACAATCAGGTGGCGTTCACTACTGATCC
GACCGCTGGGCGTTCATCTCAGTACTGTACTGACGTAGCAAAAGCTCTGAATGCT
CCGATTTTTCATGTTAATGGGGATGATATGGAGGCTGTGGTGCATGTATTTGAGCTT
GCGGCTGAGTGGCGGCAGACGTTTCATAAAGATGTGGTTGTTGATGTGGTATGTTA
CCGCCGATTTGGGCATAATGAGATGGATGAACCCTCTTTCACGCAACCGAAGATG
TACCAGGTGATTCGTAGTCACCCAAGTGCTCTTCAAGTGTACCAGAAGAAATTGG
TAGAATCGGCCCGCATCACCAAAGAGGAGATTGATGGGATACACCGTAAGGTTAG
TTCCATTTTGAATGAAGAGTACAACAGCAGCAAGGACTGCATTCCGAAGAGAAG
AGACTGGCTCTCGGCATATTGGGCGGGTTTTAAATCCCCGGAGCAGATATCAAGG
ATCCGCAATACTGGGGTTAAGCCTGAGATTCTGAAGCGCGTGGGGGAAGCTATAA
CTTCTCTGCCGGAGAAGTTTAAGGCTCATAAAGTGTTGAAGAGGATTTTTGAGGA
GCGATCTCAGATGATTCAGACAGGGGAAGGCGTTGACTGGGCGGTCGCGGAGGC
GCTTGCCTTTGCTACACTCCTGGTGGAGGGTAATCACGTTAGATTGAGTGGACAA
GACGTTGAAAGAGGAACTTTCAGCCACCGACATTCTATCATCCACGATCAAGAGA
CGGGGGAAATATACTGCCCGCTGGACCATGTTATGATGAATCAGAACGAGGAGAT
GTTTACCGTCAGCAACAGCTCATTGTCTGAATTTGGGGTTCTGGGGTTTGAGCTCG
GGTACTCCATGGAAAACCCTAATTCTCTAGTGCTCTGGGAAGCCCAATTTGGGGAT
TTTGCCAACGGTGCCCAAGTGATTTTTGACCAGTTTTTGAGTAGCGGGGAGTCGA
AATGGCTAAGGCAAACCGGCCTTGTTGTGTTGCTTCCACATGGGTATGATGGCCA
AGGCCCTGAACACTCTTCTGCGAGATTGGAGCGTTTTCTGCAGATGAGCGATGAT
AATCCTTACGTGATCCCTGAGATGGAGCCCACCATGAGGAAACAGATACAAGAG
TGCAATTGGCAAGTAGTCAACGTTACAACACCGGCTAATTACTTCCATGTACTGCG
GCGCCAGATCCACAGAGAATTCAGGAAACCACTGATTGTAATGGCTCCGAAGAA
TCTTCTGAGGCACCCCAGGTGCAAGTCAAACCTCTCAGAATTCGATGACGTTCAA
GGTCATCTGGGTTTCGACAAACAAGGAACTCGGTTCAAACGCCTCATCAAGGAC
CAAAGTGACCACTCTGATCTCGAAGAGGGCATCAGGCGTCTGGTCCTCTGCTCCG
GAAAAGTTTATTATGATCTTGATGAAGAGAGGAAAGTGGCAGAGGCCAAGGATG
TGGCGATCTGTAGAGTGGAGCAGCTCTGCCCCTTCCCTTATGACCTTGTCCAGCGG
GAGCTCAAACGATATCCTAATGCGGAAGTGGTGTGGTGTCAGGAAGAACCGATG
AACATGGGAGCATATTATTACATTAACCCTCGCCTCTCCACCGCCATGTCAGCTCT

TGGCAGGTCCTCAGTCAAAACTGTCAAATACGTTGGCCGGCCGCCATCAGCCTCC
ACCGCCACTGGCTTCCTCGGAGTCCATCAGAAAGAGCAGAAGGACCTTGTCCAC
AAGGCCATCGGCCCTGATCCCATCACATTCCCTCACTAA

LtSDH1
ATGGAGGATGGAACCCTTCACAGGTTCCGTGCGGCCAATACAATTCTAGCCACAG
GGGGCTATGGCAGAGCCTACTTTTCCGCAACTTCTGCTCACACCTGCACCGGAGA
TGGGAATGCCATGGTTTCTCGTGCTGGTCTACCTCTTCAGGATCTTGAGTTTGTACA
GTTCCACCCGACGGGAATATATGGGGCTGGGTGCCTGATTACGGAAGGATCTCGT
GGGGAAGGTGGTATTCTCAGGAATAGCGAAGGGGAGCGGTTTATGGAAAGATAT
GCTCCTACGGCCAAAGATCTTGCATCAAGAGACGTTGTTTCGAGATCCATGACTAT
GGAAATAAGAGAAGGCCGTGGTGTAGGACCACTGAAGGATCACATATATCTTCAT
CTTAATCACTTGCCGCCGGATGTGCTGAAGGAAAGACTGCCAGGCATATCTGAAA
CTGCCGCCATCTTCGCTGGAGTTAATGTAACCAAGGAGCCGATTCCCGTTCTACCA
ACTGTACACTATAACATGGGTGGTATTCCTACTAATTACCATGGGGAGGTTGTTCAT
GTCAATGGAGACGACGCCAACGCCGTAGTTCCTGGTCTAATGGCAGCTGGTGAGG
CAGCTTGTGCATCAGTTCATGGCGCAAACAGGCTTGGTGCAAACTCTTTGCTGGA
CATCGTTGTCTTTGGTCGAGCCTGTGCGAACAGAGTTGCAGAAATATCCAAACCA
GGCGACAAGCTGAAACCTCTTGAAAGGACTGCGGGAGAGAAGACCATTGCCTGG
TTAGATAAGCTGCGGAATGCTAATGGTTCACTAACCACTTCAAAAATCAGGCTTA
ACATGCAACGGGTGATGCAAAATAATGCCGCTGTTTTCCGCACTCAGGAGACTCT
TGAAGAAGGTTGTAAGTTAATCAATCAAGCTTGGGAGAGTTTCCAGGACGTCCAG
ATCCGTGATCGAAGTCTCATATGGAATTCGGACTTAATTGAGACCATAGAGTTGGA
AAACCTTCTCATCAATGCGTGTATTACCATGCATTCTGCCGAGGCAAGGAAAGAA
AGTAGAGGCGCTCATGCCCGCGAAGACTTCACTAAAAGAGACGACGAGAAATGG
ATGAAGCATTCTCTGGGGTACTGGGAAGATGAGAAGGTTCGACTGGACTATAGGC
CAGTCCATATGGATACCTTGGATGATGAAATCGAGTCATTCCCGCCAAAGGCACG
CGTTTACTGA

LtFUM1
ATGGGGATGCTAGCAGCGTCGCGTCGGATTTCATGTCGGATGAACAGTGGAGTAT
CTATGCTAGCTGGAATTCTTCAATCAGCGTACGTAAGGTCGATTTCCTCGGCGTTC
AGGGAGGAAAGGGACACGTTCGGGCCTATACAAGTGCCGGCGGACAGGTTATGG
GGAGCTCAAACTCAAAGATCGCTGCAAAACTTTGACATCGGTGGTCAATGCGAG
CAGATGCCGTTGCCAATCATTCGTGCATTTGGCATTCTGAAGAAATGCGCCGCAA
AGGTGAATATGGAATATGGTCTTGATCCGATTATCGGAAAAGCGATAATCCAAGCT
GCCCAAGAGGTGGCTGAAGGAAAGCTCAATGATCATTTTCCTCTAGTGATTTGGC
AAACCGGCAGCGGGACCCAGAGCAACATGAATGCCAATGAGGTGATAGCCAATC
GGGCGGCAGAGATTCTAGGGCATAAACGCGGAGAGAAGCTTGTGCATCCAAATG
ATCATGTCAACAGATCACAGTCCTCAAACGACACATTTCCAACTGTAATGCATATT
GCGGCTGTATTAGAGATAAAAAATATTCTGCTCCCTGGTTTATCGGGATTGCACTCT
GCCCTCTTTCAGAAGTCTCATGAGTTTAGAGATATCATCAAGATAGGGCGCACGC
ATACACAAGATGCCACTCCCTTAACTCTTGGGCAGGAGTTTAGTGGCTATTGTACT
CAGGTAGAGTATGGAATCGACAGAGTATCAGGTACTTTGCCTCACATGTATCAGCT

GGCCCAGGGTGGAACTGCCGTAGGAACTGGTTTGAACACCAAGAAAGGATTTGA
TGTGAAGATTGCTGCTGCAGTGGCAGAAGAAACTAGTCTGCCATTTGTCACGGCA
GAGAACAAATTCGAAGCGTTGGCTGCTCATGACGCTTTCGTGGAAACCAGTGGG
GCATTGAACACCGTCTCTGCTTCCTTAATAAAGATCGCGAACGACATACGGCTTCT
AGGAAGTGGTCCTCGCTGTGGACTAGGAGAACTCATTCTCCCCGAAAATGAACC
AGGCAGTAGTATCATGCCAGGCAAAGTAAACCCAACACAGTGTGAAGCTCTCAC
GATGGTCTGTTGTCAGGTAATGGGTAATCATGTGGCCATAACTATTGGTGGGTCTA
ATGGGCACTTCGAGCTGAATGTCTACAAGCCGCTCATCGCCAGTAACTTGATCCG
ATCGGTGAGGTTGCTAGGCGATGCAGCGTCGTCCTTTGAGAAGAACTGTGTGAGC
GGTATTCGGGCTAACAATCAGAGGATTTCGAAGCTGTTGCATGAGTCCTTGATGCT
GGTCACCTCCTTGAATCCTAAAATCGGCTATGACAAGGCTGCTGCAGTAGCTAAG
AAAGCTCACAGGGAGGGTACTACCCTGAAGGATGCTGCACTCGGCCTTCAAGTC
CTCACCGCTCAAGAATTCGATGAGCTAGTGGTCCCAGAGAAGATGATTGGCCCCT
CTGACTGA

LtMDH1
ATGATCAAGAGGCGCTTCTACAAGTTGGAGCATTCCGCTCGCGACGGTTCTTCGT
CCTCGTCTTCGGAGTCTGAAGACGAAGTGGGAGAACGAGAGGAGGAGTCGCATG
GTGAAGAAGAGGTCGAGGATAAGGAAGTGACTGCTGAAAAATGGCGGAAGCAG
TTCTCTGTGTCTGACGACGCTTCATCTGGGTCTGGTTATGAGAGCGAGGAGAGCTC
ACGGAATGACATCAGTTGTGACGCTACAGGCTTACCACTTAATGATAACGACGAT
GACGAACGACATCAAGGTCAAACTAAAAATGAGGGCAAAATCAATGGCTCGGA
AGATCAAAGCCAGTCTGAGATGGATTACTACGTGCTGAAGCACAAGTCTGTATTC
AAGTGTAGGATCTGCCCTAGAATCATCTGCTTATCTGAGAACACCCTTCTGGAACA
TCTTAAATCCAAGAGACATGCCAGATCTAAGAAGTTATTGAAGGAGGGAAGACTC
AAGCTGATGCTGAACAGCGATGGAGAGATTGAAGAGGATCAAGAGACCCACGC
CGAAAGGCACGCCAGGACTATCGCTCTCGCTCAGGTTCCCATTTATTGTGTTAAAA
GTAATGATTATTTAATTTAG

LtCS1
ATGGTGTTCATGCGGAGTGTTTCGGTACTTTCCAAGATCCGATCCCGCCTGGTGCA
ACAATCGGCGACTTTAGGCTCTGTCAGATGGCTTCAGATCCAGAGTTCTTCTGATC
TAGATATTCGTGCGCAATTGAAGGATCTTATTCCAGAGCAACAGGAGCGCTTGAA
GAAATTGAAATCAGAGCACGGTAAAGTTGAGTTAGGAAAGATCACTCTTGACATG
GTAATTGGTGGAATGCGAGGAATGACTGGATTGCTGTGGGAAACTTCGTTGCTTG
ATCCTGAAGAGGGAATCCGTTTTAGAGGTCTTTCTATTCCTGAATGTCAGAAAGTG
CTGCCTGCTGCAGTTCCTGGTGGTGAACCATTGCCCGAAGGCCTTCTGTGGCTTCT
TTTGACCGGAAAGGTTCCTAATAAGGATCAAGTCATTTCTTTATCCAAGGAGCTGC
AAGCACGTTCTTCAGTTCCAGATTATGTTTACAAGACTATTGATGCACTCCCTATAA
CCTCTCATCCAATGACACAGTTTGCATCTGGTGTCATGGCACTTCAGGTTGAAAGC
GAATTTCAGAAGGCATACGAGAAAGGAATATCAAAAACAAAGTATTGGGAGCCG
ACCTTTGAAGATTCTCTTAACCTGATAGCCCGACTTCCTGCAGTGGCAGCTTATGT
CTATAGAAGAATATACAAAAATGGACAGATCATACCTGCTGATGAATCATTGGACT
ATGGCGGAAATTTCTCCCACATGCTTGGGTTTGATGATCCCAGAATGCTGGAGCTG

ATGAGACTATATGTTACTATCCACAGTGATCATGAAGGGGGTAATGTTAGCGCCCA
TACTGCTCATCTGGTTGGGAGTGCACTTTCAGACCCATATCTTTCATTTGCAGCTGC
ATTAAATGGTTTAGCAGGCCCGCTTCACGGGTTGGCTAATCAGGAAGTACTGCTAT
GGATCAAATCTGTCGTAGAAGAATGTGGGGAGAACATTACAACCGACCAACTGA
AGGAGTATGTGTGGAAGACCTTAAACAGTGGCAAGGTTGTTCCCGGATACGGTCA
CGGTGTTCTTCGCAAAACAGATCCAAGATACGTGTGCCAAAGGGAGTTTGCTCTC
AAGTACTTGCCAGATGACCCACTATTCAAGCTGGTCTCAAAGGTTTACGATGTTGT
TCCCCCAATACTCACTGAACTTGGCAAGGTTAAAAACCCGTGGCCAAATGTAGAT
GCTCACAGTGGAGTCTTGTTGAATTACTTCGGCTTGTCGGAAGCTAGGTACTTCAC
CGTTCTCTTTGGTGTCTCGAGAAGCATTGGCATTTGCTCCCAGCTGATATGGGACC
GGGCGTTAGGACTGCCACTTGAACGCCCTAAGAGTGTGACGATGGATTGGGTGG
AAAGCTTCTGCAAGAAGGCTGCTTGA
